# Synthesis and supramolecular properties of regioisomers of mononaphthylallyl derivatives of γ-cyclodextrin

**DOI:** 10.3762/bjoc.13.248

**Published:** 2017-11-27

**Authors:** Markéta Bláhová, Sergey K Filippov, Lubomír Kováčik, Jiří Horský, Simona Hybelbauerová, Zdenka Syrová, Tomáš Křížek, Jindřich Jindřich

**Affiliations:** 1 Department of Organic Chemistry, Faculty of Science, Charles University, Hlavova 8, 128 43 Prague 2, Czech Republic; 2 Institute of Macromolecular Chemistry, Academy of Sciences of the Czech Republic, Heyrovsky Sq. 2, 162 06 Prague 6, Czech Republic; 3 Institute of Biology and Medical Genetics, First Faculty of Medicine, Charles University, Albertov 4, 128 01 Prague 2, Czech Republic; 4 Department of Teaching and Didactics of Chemistry, Faculty of Science, Charles University, Hlavova 8, 128 43, Prague 2, Czech Republic; 5 Department of Analytical Chemistry, Faculty of Science, Charles University, Hlavova 8, 128 43, Prague 2, Czech Republic

**Keywords:** γ-cyclodextrin, naphthylallyl derivatives, regioselective alkylation, supramolecular properties, synthesis

## Abstract

Monosubstituted derivatives of γ-cyclodextrin (γ-CD) are suitable building blocks for supramolecular polymers, and can also serve as precursors for the synthesis of other regioselectively monosubstituted γ-CD derivatives. We prepared a set of monosubstituted 2^I^-*O*-, 3^I^-*O*-, and 6^I^-*O*-(3-(naphthalen-2-yl)prop-2-en-1-yl) derivatives of γ-CD using two different methods. A key step of the first synthetic procedure is a cross-metathesis between previously described regioisomers of mono-*O*-allyl derivatives of γ-CD and 2-vinylnaphthalene which gives yields of about 16–25% (2–5% starting from γ-CD). To increase the overall yields, we have developed another method, based on a direct alkylation of γ-CD with 3-(naphthalen-2-yl)allyl chloride as the alkylating reagent. Highly regioselective reaction conditions, which differ for each regioisomer in a used base, gave the monosubstituted isomers in yields between 12–19%. Supramolecular properties of these derivatives were studied by DLS, ITC, NMR, and Cryo-TEM.

## Introduction

Cyclodextrins [1] (CDs) are cyclic oligosaccharides with a cone-shaped cavity formed by α-1,4-linked D-glucopyranose units. The most widely used CDs are α-, β-, and γ-CD with 6, 7 or 8 glucose units, respectively. Both chemically modified and native CDs are used in numerous applications, e.g., in separation methods [2–3] or in the pharmaceutical industry [4–5]. CDs are well-known as host molecules for various guest substances in aqueous solutions [4]. Derivatives of CDs are attractive building blocks for various types of supramolecular structures [6–7]. Necessary non-covalent interactions depend on the type and derivatization of CD, on lipophilicity, shape, and size of the guest molecule, and on conditions such as temperature, pH, or solvent used [8]. In supramolecular polymers [9] multifunctional molecules are assembled in a regular manner. To avoid branching, monosubstituted CDs are required for a linear supramolecular polymer of CDs with guest groups attached.

Monosubstituted derivatives of CDs [10] include three regioisomers, namely 2^I^-*O*-, 3^I^-*O*-, and 6^I^-*O*-substituted ones. To separate pure regioisomers from their mixture is tricky. Their direct regioselective synthesis is possible, although not easy, because hydroxy groups at different positions of CD have different properties; the hydroxy group at position 6 is the most nucleophilic and basic one, the hydroxy group at position 2 is the most acidic, and the group at position 3 is most sterically hindered. Therefore, regioselective monosubstitution to positions 2 and 6 can be controlled by the amount and strength of the base used in the reaction, whereas monosubstitution at position 3 can be achieved by complexation of the alkylation agent to the CD cavity and orientation of the agent´s reactive center towards the 3-OH group [11–13].

Here, we report the preparation of regioisomers of novel mononaphthylallyl γ-CD (NA-CD), i.e., 3-(naphthalene-2-yl)prop-2-en-1-yl γ-CD, which is interesting for several reasons. Compared to α- or β-CD, the number of publications dealing with γ-CD is quite small, e.g., [14–15]. The naphthyl group is well known for its ability to make inclusion complexes with CDs [16]. The allyl connecting linker is relatively rigid and should support the formation of supramolecular polymers of CD–guest type [17]. Moreover, the double bond of the allyl group allows a subsequent conversion of these compounds to other regiospecifically monosubstituted γ-CD derivatives. Naphthylallyl derivatives of any CD have not been described yet.

Supramolecular properties of the prepared derivatives were studied by a set of methods allowing characterization of supramolecular behavior at various levels – from binary complexation, over supramolecular oligomers to large assemblies; namely by isothermal titration calorimetry (ITC) [16], ^1^H nuclear magnetic resonance spectroscopy (^1^H NMR), dynamic light scattering (DLS) [18–21] and cryo-transmission electron microscopy (Cryo-TEM) [22–24].

## Results and Discussion

### Synthesis

We present two approaches for the synthesis of naphthylallyl (NA) derivatives. The first approach is a cross-metathesis of peracetylated allyl derivatives of γ-CD with 2-vinylnaphthalene. The main advantage of this procedure is that a pure regioisomer of the product is obtained due to the regio-purity of the starting material. Unfortunately, this reaction is quite low-yielding, and the isolation of the products is hard to perform. Moreover, the starting compound for this reaction has to be prepared from γ-CD in two steps involving complicated chromatographic separations. Thus, the overall yields of the pure NA regioisomers were only 2–5%. Therefore, we turned our focus to developing another method for the preparation of naphthylallyl derivatives, which is based on a direct alkylation of γ-CD with 2-(3-chloroprop-1-enyl)naphthalene.

#### Cross-metathesis

**Scheme 1:**
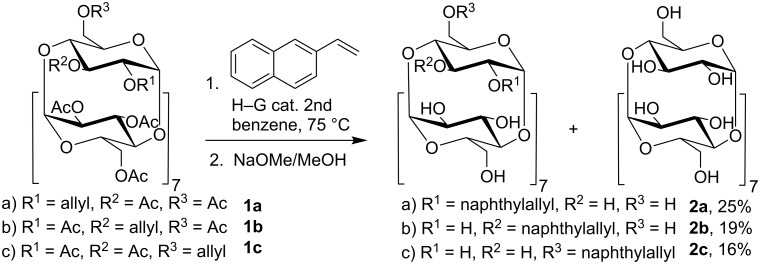
Preparation of 2^I^-*O*-, 3^I^-*O*- and 6^I^-*O*-naphthylallyl derivatives of γ-CD by cross-metathesis.

Mono 2^I^-*O*-, 3^I^-*O*- and 6^I^-*O*-naphthylallyl-γ-CDs (2-*O*-NA-γ-CD, **2a**, 3-*O*-NA-γ-CD, **2b**, 6-*O*-NA-γ-CD, **2c**) were prepared from peracetylated *O*-allyl derivatives [14] by cross-metathesis with 2-vinylnaphthalene (Scheme 1) in the presence of the Hoveyda–Grubbs 2nd generation catalyst. After deacetylation, column chromatography afforded the naphthylallyl derivatives in yields between 16–25%, which is comparable to the results of other metathesis reactions of allylated CDs [11,25]. In all cases, only the isomer with *E* configuration of the NA double bond was observed by ^1^H NMR. Reactions were performed in benzene at 75 °C or dichloromethane at 45 °C, the latter giving lower yields. Prior to the isolation, it was necessary to perform deacetylation of the reaction mixture because the product had the same *R*_f_ as the starting material. After the deprotection, a substantial amount of the pure γ-CD was isolated as the only byproduct containing CD. Apparently, a concurrent reaction – the cleavage of allyl ether – also took place. The use of similar ruthenium complexes as catalysts for the cleavage of allyl ethers and allyl esters was described before by Tanaka et al. [26–27].

#### Naphthylallylation

**Scheme 2:**
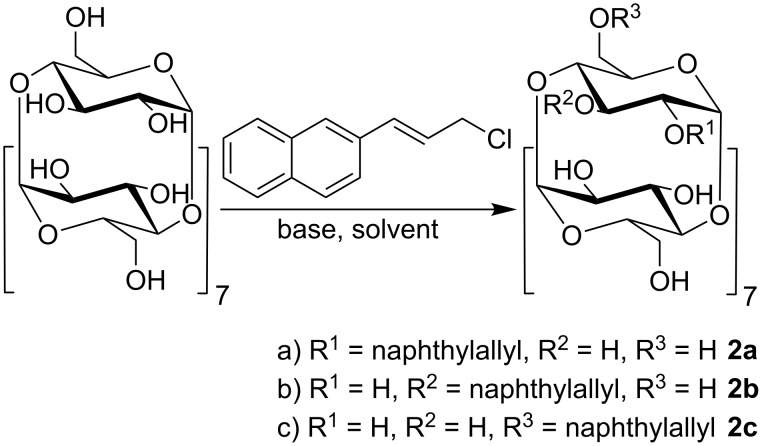
Preparation of 2-*O*-, 3-*O*- and 6-*O*-NA derivatives of γ-CD by direct alkylation (see Table 1 for the yields and conditions).

The same naphthylallyl derivatives of γ-CD **2a**–**c** were also prepared by direct alkylation of γ-CD with 2-(3-chloroprop-1-enyl)naphthalene [28] as the alkylation reagent (Scheme 2). This reagent is easy to prepare on a large scale without a need of purification by chromatography. Yields of the NA regioisomers **2a**–**c** were, after isolation by column chromatography, in the range 1–24% (Table 1). The optimization experiments for this procedure, followed by TLC, revealed that the products are formed in all cases, but the yields and regioselectivity depend substantially on the amount and the type of a base. Anhydrous DMSO was selected as a solvent because it often proved to be superior to DMF in our previous experiments in alkylations of CDs.

**Table 1:** Preparation of 2-*O*- (**2a**), 3-*O*- (**2b**) a 6-*O*- (**2c**) NA derivatives of γ-CD by direct alkylation with 2-(3-chloroprop-1-enyl)naphthalene.

entry	base, solvent	yields^a^ (%)		

		**2a**	**2b**	**2c**
		
1	1.5 equiv EtONa, DMSO	1	19	–
2	4 equiv EtONa, DMSO	6	<1	–
3	15 equiv EtONa, DMSO	15	–	23
4	1.5 equiv LiH, LiI, DMSO	3	24	–
5	15 equiv LiH, LiI, DMF	13	<1	–
6	10 equiv NaOH, DMSO	14	<1	–
7	15 equiv NaOH, DMSO	16	–	5
8	20 equiv LDA, DMSO	11	<1	–
9	15 equiv NaH, DMSO	<1	–	12

^a^Isolated yields; <1 no isolated yield, – not observed.

Direct alkylation methods have been studied in CD chemistry over last twenty years [11,14,29–34]. As mentioned before, it is possible to direct the alkylation to the specific position just by changing the amount of a base used for the reaction. For example, the alkylation of the secondary rim of CDs can be achieved by using an equimolar amount of the base. In these cases, perdominantly the 2-*O*-substituted product is formed, but a small amount of the 3-*O*-derivative can be present in the reaction mixture as well. However, in the case of NA derivatives, the 3-*O*-derivative is formed preferentially (Table 1, entries 1 and 4), which is the same outcome we have observed earlier in the case of the 3-*O*-cinnamyl-β-CD [35].

During the attempts to prepare the 6-*O*-substituted derivative, we came across unusual changes in the regioselectivity of the substitution. In previous publications, we have shown that the substitution to the position 6 can be achieved by using an excess of NaOH (30 equiv) in aqueous solvents [11,30,35]. Unfortunately, this method is not applicable for the reaction described in this paper because the alkylation reagent hydrolyzes too fast and the desired product is not formed. Attempts to use 30 equiv of a base in nonaqueous solvents also lead only to decomposition of the alkylation agent. Therefore, a new set of optimization experiments for developing suitable reaction conditions for the preparation of the 6-*O*-derivative had to be made. Different amounts (1.5–20 equiv) and types of bases were used. Interestingly, the highest yields of 6-*O*-isomer gave 15 equiv of a base; however, the same conditions gave unexpectedly also the 2-*O*-derivative. In experiments with more than 1.5 equiv of a base (Table 1, entries 2, 5, 6, 8), the 2-*O*-derivative was obtained almost regiospecifically, but entries 3 and 7 gave mixtures of 2-*O*- and 6-*O*-derivatives. However, the 6-*O*-derivative can be obtained regioselectively with 15 equiv of NaH (Table 1, entry 9). The robustness of this method was confirmed by an experiment using just 12 equiv of NaH resulting in practically the same outcome.

The 2-*O*-, 3-*O*- and 6-*O*-NA regioisomers of γ-CD offer a significant advantage – they are separable by column chromatography in the elution mixture PrOH/H_2_O/NH_3_, which cannot be achieved for other monoderivatives of α-, β- and γ-CD with smaller substituents such as allyl and propargyl having similar *R*_f_ and must be peracetylated prior to other separation processes.

#### Dynamic light scattering (DLS)

**Figure 1:**
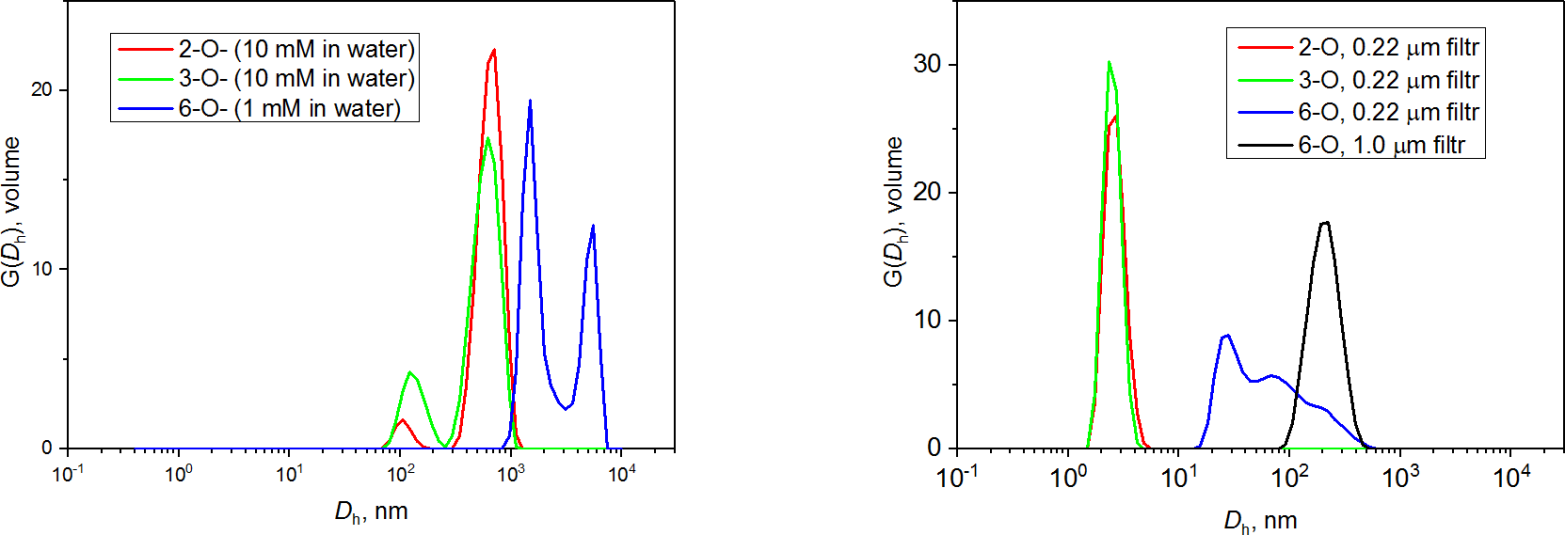
Volume-weighted distribution functions for water solutions of 2-*O*- (**2a**), 3-*O*- (**2b**), and 6-*O*- (**2c**) NA-γ-CD regioisomers before (left) and after (right) filtration.

The supramolecular properties of all regioisomers of NA-γ-CD (2-*O*-, 3-*O*-, 6-*O*-) were investigated by DLS. We analyzed the dependence of self-assembling of the isomers on external stimuli such as agitation and temperature. The 3-*O*-isomer **2b** shows co-existence of two types of aggregates: large aggregates (around 600 nm in diameter) and smaller aggregates (around 140 nm in diameter, Figure 1). Aggregates formed by 2-*O*-isomer **2a** were similar to those of the 3-*O*-sample. On the other hand, the 6*-Ο-*isomer **2c** produced different results. Since the measurement of this isomer was complicated due to its low solubility in water, the sample was first dispersed in water and then heated to 120 °C in a sealed ampule. The solution became turbid after cooling, which manifests the presence of large particles. Indeed, the DLS experiments revealed large particles of the 6-*O*-sample with sizes larger than 1 μm, exceeding the limit of the instrument used.

To assess the influence of filtration on the self-assembly of naphthylallyl regioisomers, samples were then passed through 0.22 μm pores. The volume-weighted DLS distribution function of the 2-*O*- and 3-*O*-samples show a dominant presence of 2.5 nm particles immediately after the filtration. The sample of the 6*-Ο-*isomer showed the distribution of sizes ranging from 30 nm to 200 nm after filtration through 0.22 μm pores and 200 nm aggregates after filtration through 1 μm pores. In either case, the 6-*O*-isomer formed aggregates with a broader distribution of sizes than the 2-*O*- and 3-*O*-isomers and no 2–3 nm particles were observed in the sample. Nevertheless, small particles still may be present in solution because the intensity of light scattered by hard spheres is proportional to *R*^6^ (*R* being the radius) which means that large particles scatter significantly more light than the same mass of small particles. Thus, a fairly small amount of large aggregates might dominate the scattering making the contribution of small particles negligible.

The long-time evolution of all the isomers after filtration was evaluated according to the approach of Bonini [22] and González-Gaitano [21]. The distribution function of the 3-*O*-isomer shows the presence of a dominant mode with 2.5 nm particles immediately after the filtration, which stays intact throughout the experiment. A minor fraction of larger aggregates is formed in solution shortly after filtration whose size continuously fluctuate (Supporting Information File 1, Figure S5), which could be easily monitored by scattering intensities behavior. The evolution of the 2-*O*-isomer in time has also been investigated, but no trend was observed in this case (Supporting Information File 1, Figure S6). The long-time evolution of the 6-*O*-filtered sample revealed that the size of particles continuously increased with time (Supporting Information File 1, Figure S6), starting from 2 nm particles appearing immediately after the filtration and reaching larger particles that grew in the sample standing overnight.

**Figure 2:**
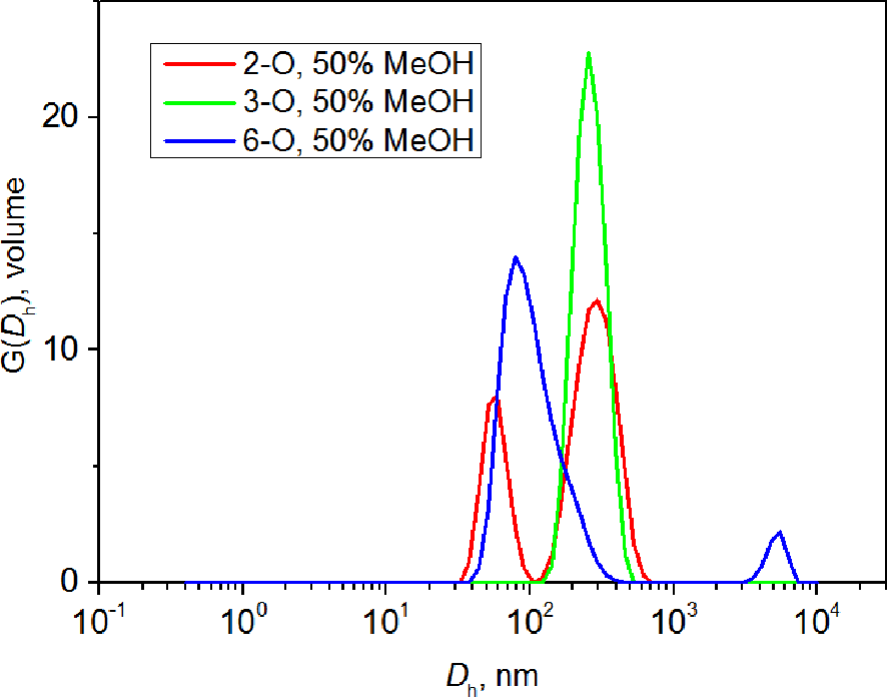
Distribution functions for 2-*O*- (**2a**), 3-*O*- (**2b**), and 6-*O*- (**2c**) NA-γ-CD regioisomers in 50% MeOH (v/v).

Subsequently, the effect of different solvents on the size of aggregates was investigated by a method proposed by us for monocinnamyl-α-CD (Cin-α-CD) derivatives [20]. Two other water-solvent mixtures were tested. In 50% MeOH (v/v) solution 2-*O*- and 3-*O*-isomer formed aggregates with diameters around 200 nm, but the 2-*O*-derivative contained 50 nm aggregates as well. Interestingly, the 6-*O*-isomer formed aggregates with a broad size distribution with a peak at 100 nm (Figure 2).

We observed that the 6-*O*-isomer has a low solubility in water and 50% MeOH solution, but it is more soluble in 50% PrOH (v/v). Consequently, the assembling of the 6-*O*-derivative was investigated in 50% PrOH, which revealed that just 1.4 nm particles were present in the solution. This means that the 6-*O*-derivative does not form any aggregates in 50% PrOH.

**Figure 3:**
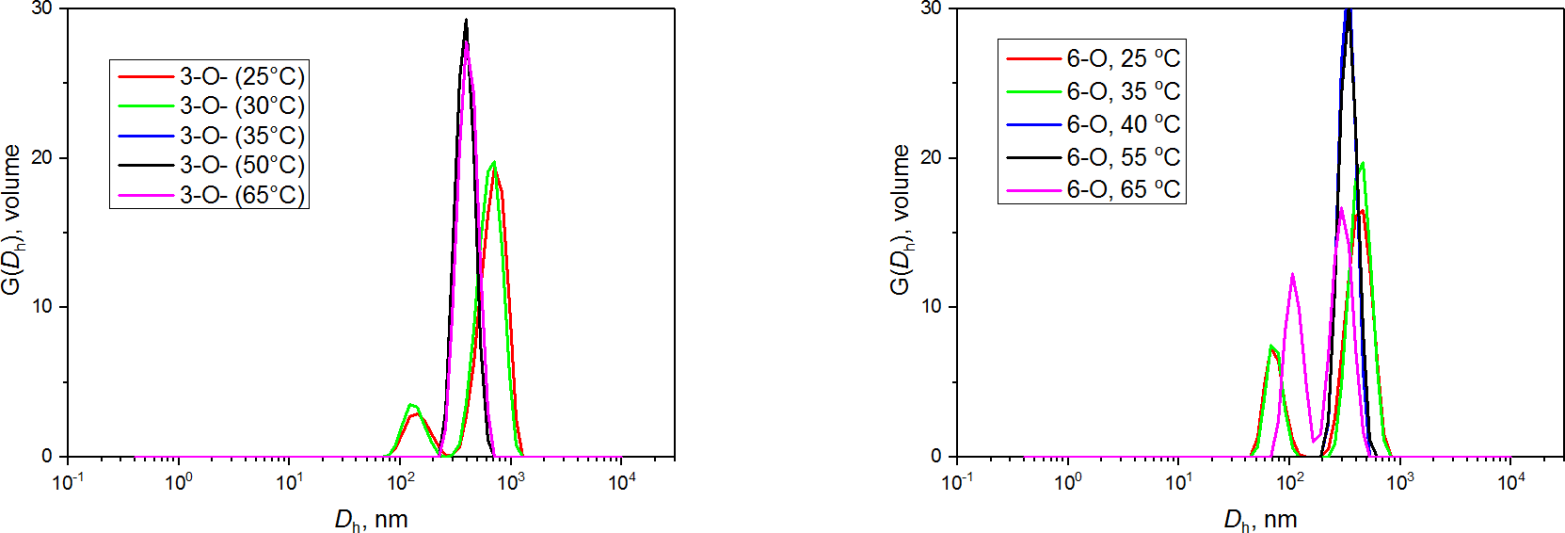
Volume-weighted distribution functions for the 3-*O*- (**2b**) and 6-*O*- (**2c**) NA-γ-CD regioisomer at different temperatures.

We investigated the temperature dependence of the aggregate size of the regioisomers in similar ways as González-Gaitano [21] and as we did for Cin-α-CD [20]. The temperature was raised from 5 °C to 65 °C (5 °C steps) across 2.5 h, which led to substantial changes in sizes of the aggregates (Figure 3). A shift of the correlation curve, caused by the change of viscosity, is noticeable for the 3-*O*-isomer (Supporting Information File 1, Figure S4). Up to 30 °C, two peaks around 150 nm and 600–700 nm were detected, whereas at 35 °C and above, just one peak around average size (300–400 nm) appeared. The increase in hydrophobic interaction at the higher temperature may be responsible for the observed behavior. The effect of temperature was not as pronounced as in the case of Cin-α-CD [20]. The sample of the 6-*O*-isomer had to be filtered through 1 μm pores because of its high turbidity. The sample was equilibrated for 3 h before the measurement. The temperature behavior of the 6-*O*-derivative was similar to the 3-*O*-isomer. At the low temperature (25 °C), two peaks are visible on the distribution function (Figure 3) corresponding to sizes of aggregates around 80 nm and 400 nm. At 40 °C, just one peak around 300 nm appeared, and at 65 °C, two peaks appeared again, around 100 nm and 300 nm. The 2-*O*-sample did not show any temperature dependence.

#### Transmission cryo-electron microscopy (Cryo-TEM)

The supramolecular properties of the individual regioisomers (2-*O*-, 3-*O*-, 6-*O*-) of NA-γ-CD **2a–c** and the dependence of the formation of the self-assembled structures on their concentration were also determined by Cryo-TEM.

**Figure 4:**
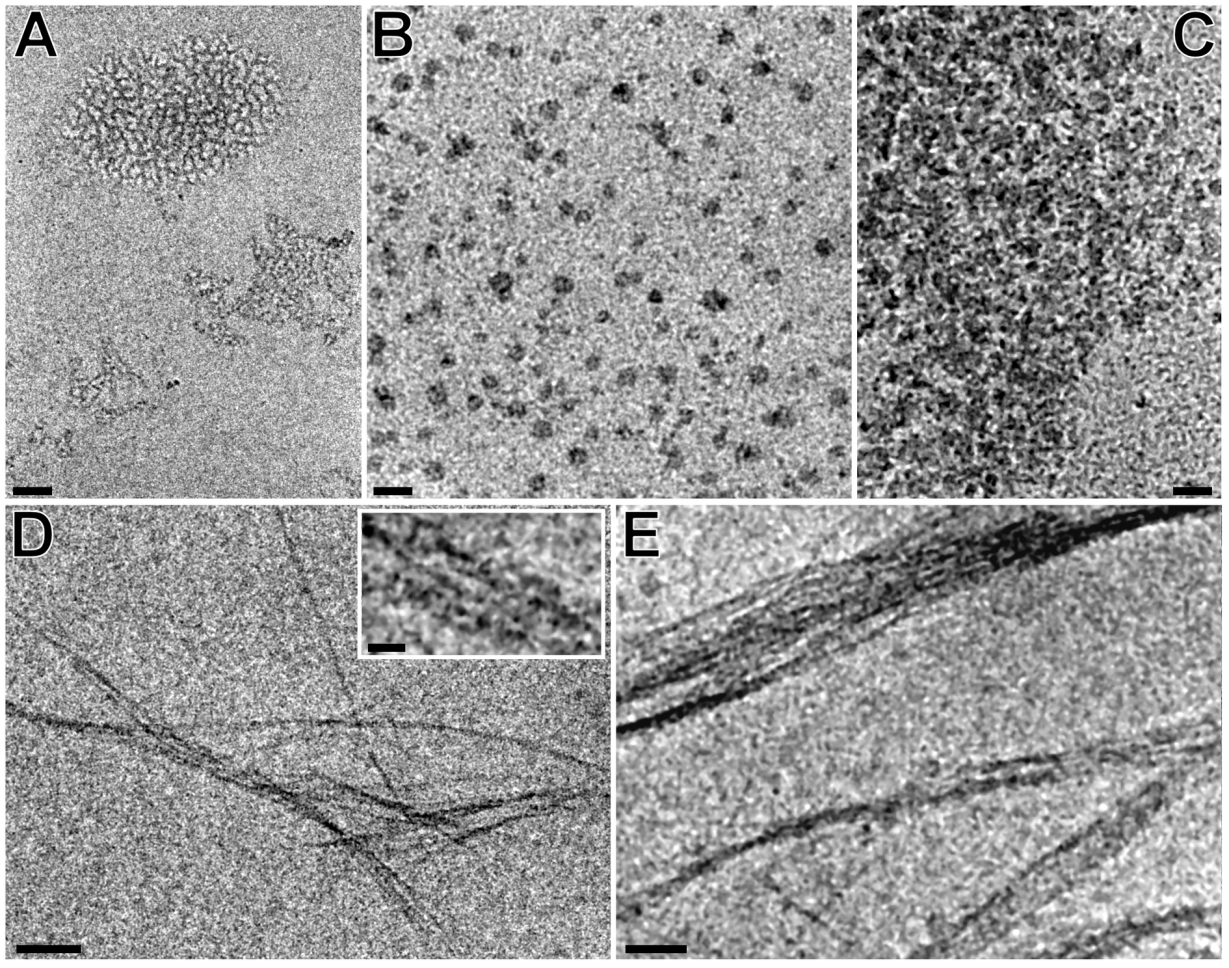
Effect of increasing concentration and sonication on the morphology of the 3-*O*-derivative **2b**. A to C: 2 mM, 20 mM, and 100 mM unsonicated specimens; D: fibers of the 2 mM specimen after sonication. Inset: high-magnification detail of the fibers; E: fibers and aggregates of the 100 mM specimen. Scale bars: A–C: 50 nm, D–E: 100 nm, inset: 20 nm.

While the 2 mM sample of the 3-*O*-derivative **2b** formed irregular clumps of fibers of the thicknesses 1–2 and 4–7 nm having a size of 50 to 100 nm (Figure 4A), the 20 mM sample contained smaller spherical clusters of size 20 to 30 nm (Figure 4B), which formed large aggregates with the size of hundreds of nm in the 100 mM sample (Figure 4C). It is evident that the method of aggregation and the morphology of the specimen vary with increasing concentration. Sonication, conducted for 2 mM and 100 mM samples, resulted in a diametrically different arrangement of the fibers (Figure 4D and E). Both sonicated solutions contained several µm long bundles of fibers with thicknesses of 1.5–10 nm; in the 100 mM sample, also particles having diameters from 30 to 120 nm were present.

**Figure 5:**
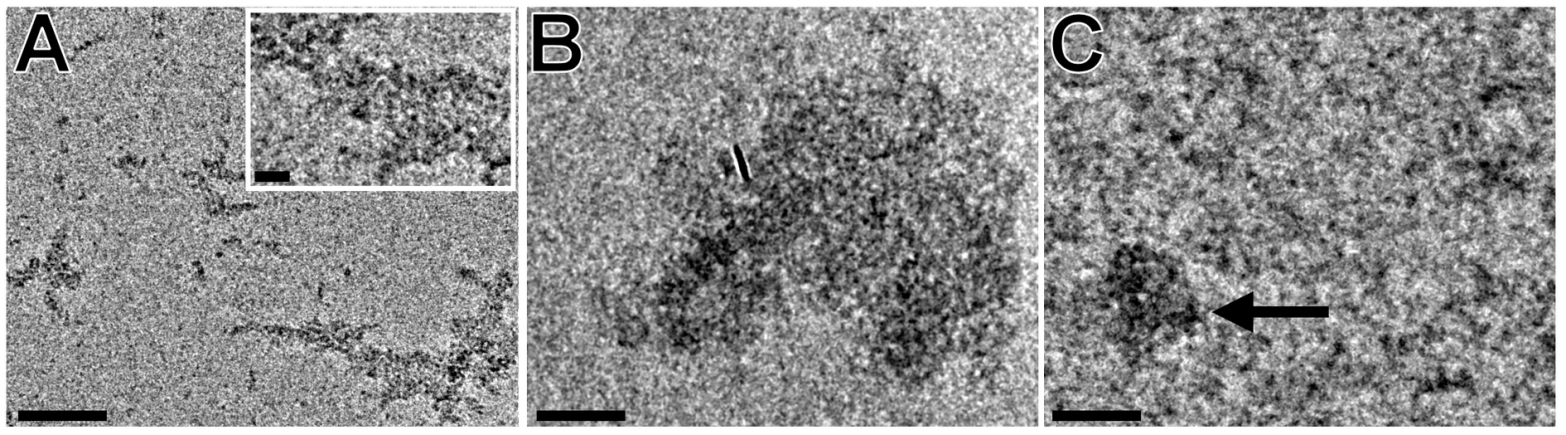
Effect of increasing concentration and sonication on the morphology of the 2-*O*-derivative **2a**. A: 2 mM unsonicated specimen; B: 20 mM unsonicated specimen; C: 100 mM sonicated specimen. The arrow indicates an aggregated particle. Scale bars: 50 nm, inset: 10 nm.

Smaller spherical particles of β-CD, which tend to combine into larger aggregates, were also observed by Bonini et al. [22], who described the presence of clusters of different sizes and shapes in one sample as well as the dependence of the cluster properties on the concentration. The 2 mM and 20 mM solution of 2-*O*-derivative **2a** formed clusters of similar shape and size as the 3-*O*-derivative **2b** (Figure 5A and B). In the 100 mM sample, the presence of large quantities of material rarely forming large particles, was evident (Figure 5C). A 10-minute sonication led just to the disappearance of the larger aggregates, no fibers in a μm scale were detected.

**Figure 6:**
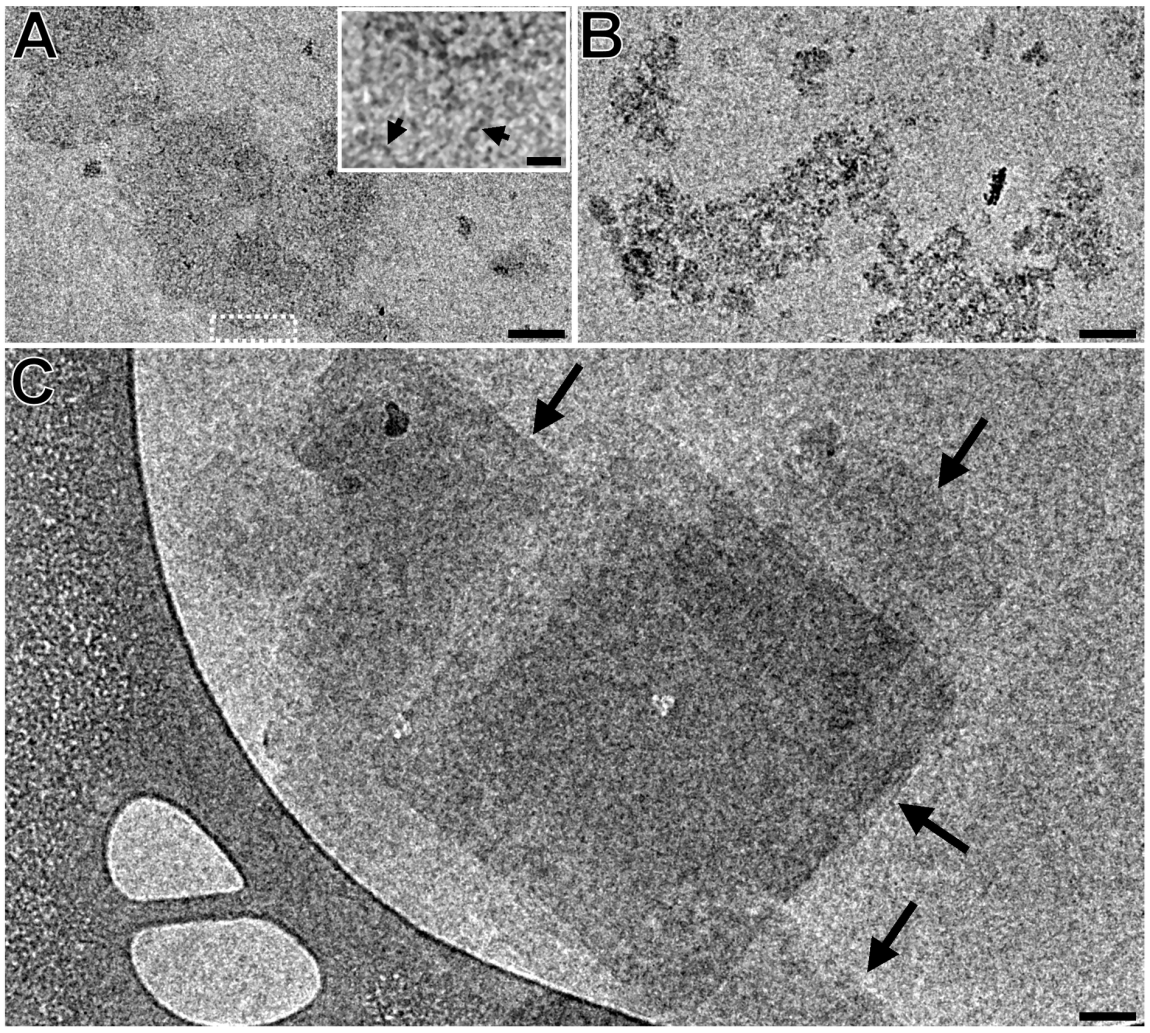
Effect of increasing concentration and sonication on the morphology of the 6-*O*-derivative **2c**. A: 0.5 mM specimen after sonication. Inset: enlarged area indicated by the white dashed rectangle. Arrows indicate the NA-γ-CD fibers; B: 5 mM sonicated specimen; C: 5 mM unsonicated specimen. Scale bars: 50 nm, inset: 10 nm.

The 6-*O*-derivative **2c**, due to its low solubility, was measured only at low concentrations of 0.5 mM and 5 mM (Figure 6A, B). In the 0.5 mM solution, only short fibers were found, whereas in the 5 mM sonicated solution, fibrous aggregates, similar to those of 2 mM unsonicated samples 2-*O*- and 3-*O*-derivatives, were observed. In an unsonicated 5 mM preparation, we observed many sheet-like structures (Figure 6C), corresponding to the observations of Bonini et al [22].

#### Isothermal titration calorimetry (ITC)

ITC experiments on NA-γ-CD cannot be carried out as classical titration experiments, i.e., by injecting the ligand solution into a solution of the receptor [36], because complementary complexing moieties are covalently bonded. Instead, the so-called dissociation [37] or release [38] experiment had to be used, in which a solution of a complex is injected initially into a pure solvent and to the dilute solution in subsequent injections (see Supporting Information File 1 for more details and the model description).

**Figure 7:**
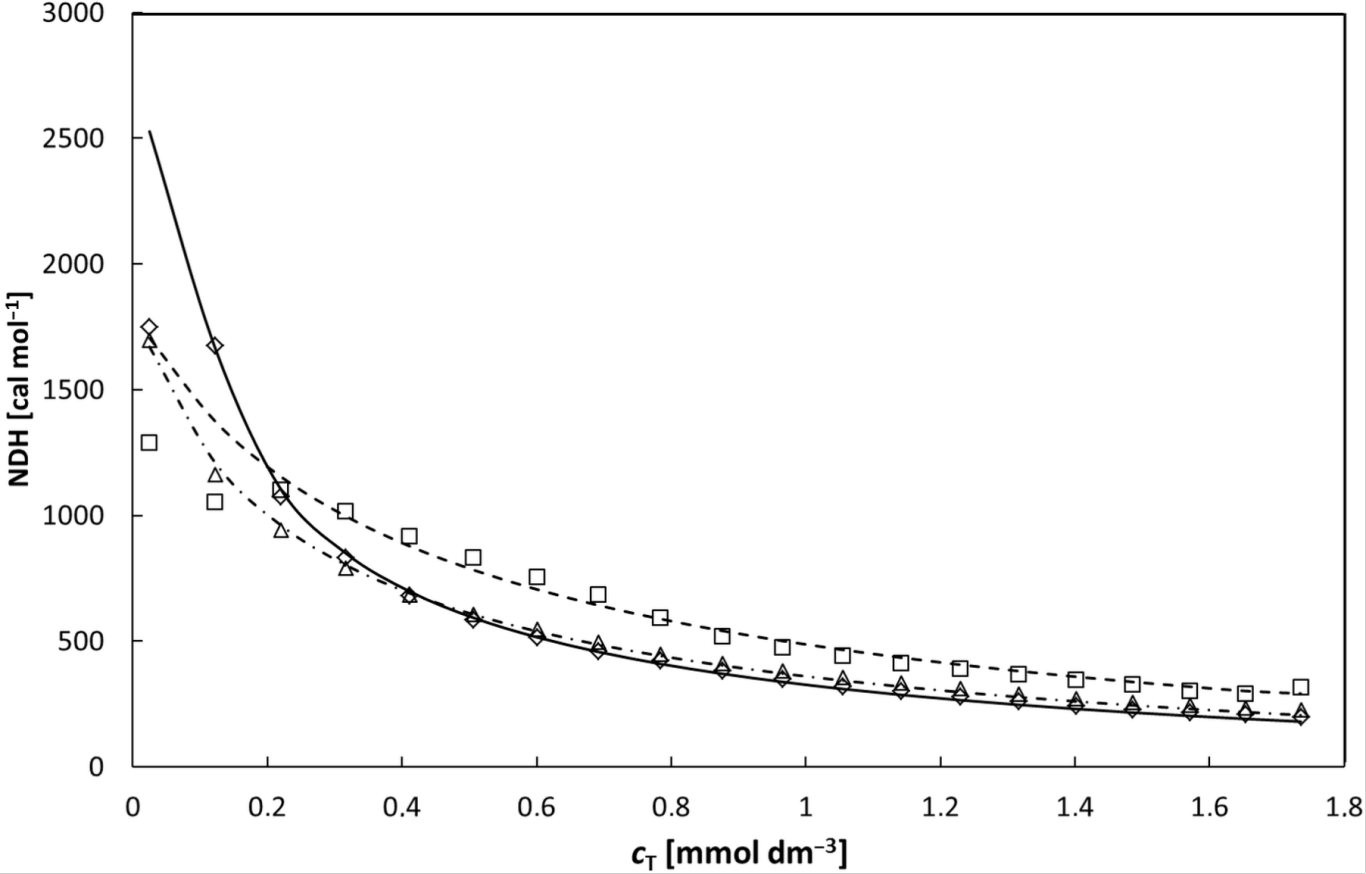
Heat change for injection per mole of NA-γ-CD added as a function of the total concentration of NA-γ-CD at 10 °C. Symbols are used for experimental values; lines for fits: 6-*O*-NA-γ-CD **2c** squares and a dashed line; 2-*O*-NA-γ-CD **2a** diamonds and a full line; 3-*O*-NA-γ-CD **2b** triangles and a dashed-dotted line.

The fit of experimental and calculated values of the normalized heat per injection (NDH) was quite satisfactory for 2-*O*- (**2a**) and 3-*O*- (**2b**) NA-γ-CD at all measured temperatures. Fits for the less soluble 6-*O*- (**2c**) were less satisfactory at 10 and 25 °C and failed at 45 °C. Results at 10 °C are shown in Figure 7.

Estimated thermodynamic parameters and average degrees of polymerization at various temperatures are collected in Table 2. The values for 6-*O*-NA-γ-CD at 45 °C are omitted and the values for 6-*O*-NA-γ-CD at 10 °C and 25 °C are given in parentheses because of their low reliability. Nevertheless, even those values, as well as those for 2-*O*-NA-γ-CD and 3-*O*-NA-γ-CD at all three temperatures correspond to values found for γ-CD and aromatic compounds [39]. Binding of the 2-*O*-regioisomer is stronger than that of the 3-*O*-regioisomer, and consequently, longer oligomers can be expected for it.

**Table 2:** Estimated thermodynamic parameters for complexation of NA-γ-CD regioisomers.

sample	*T* (°C)	*K* (dm^3^/mol)	Δ*H* (cal mol^−1^)	*T*Δ*S* (cal mol^−1^)	*n*_n_^a^	*n*_w_^b^

**2c**	10	(1215)	(−2783)	(1395)	(4.02)	(7.04)
	25	(418)	(−2439)	(1176)	(2.60)	(4.20)
	45	–	–	–	–	–
**2b**	10	2857	−2903	1795	5.87	10.74
	25	2644	−3654	1328	5.67	10.33
	45	1193	−4463	317	3.99	6.98
**2a**	10	5657	−3303	1806	8.04	15.08
	25	3489	−3667	1490	6.43	11.86
	45	1619	−4466	533	4.55	8.11

^a^Number-average degree of polymerization calculated for 10 mM solution. ^b^Weight-average degree of polymerization calculated for 10 mM solution.

The fraction of suprapolymer with polymerization degree *n* is then given for negligible cyclisation as

[1]
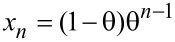


where θ is the degree of occupation, i.e., the probability that the particular group, either naphthyl or CD, is in a bound state. θ is determined by the binding constant and NA-γ-CD concentration (see Supporting Information, File 1). Number average and weight average degrees of polymerization (*n*_n_ and *n*_w_) are given by standard relations derived for polycondensation [40]

[2]
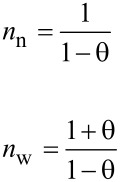


The values of *n*_n_ and *n*_w_ calculated from ITC results for 10 mM solutions of NA-γ-CD are also presented in Table 2.

#### NMR spectroscopy

The supramolecular behavior of the regioisomers was further examined by ^1^H NMR. The measurements were performed in D_2_O, where the strongest intermolecular interactions are expected. The concentration dependence of chemical shifts was taken as their indicator. The 6-*O*-isomer **2c** was excluded from these experiments because its solubility in water is very limited; samples cannot be prepared at concentrations greater than 5 mM.

Spectra for 2-*O*- (**2a**) and 3-*O*- (**2b**) derivatives were measured at concentrations of 100, 10, and 1 mM. Although these regioisomers are quite soluble in water, turbidity becomes noticeable after a longer time, and in the case of 100 mM specimens, even precipitation of a solid was observed. The turbidity caused the worse quality of ^1^H NMR spectra; however, chemical shifts and changes in the shape of the peaks are clearly visible.

**Figure 8:**
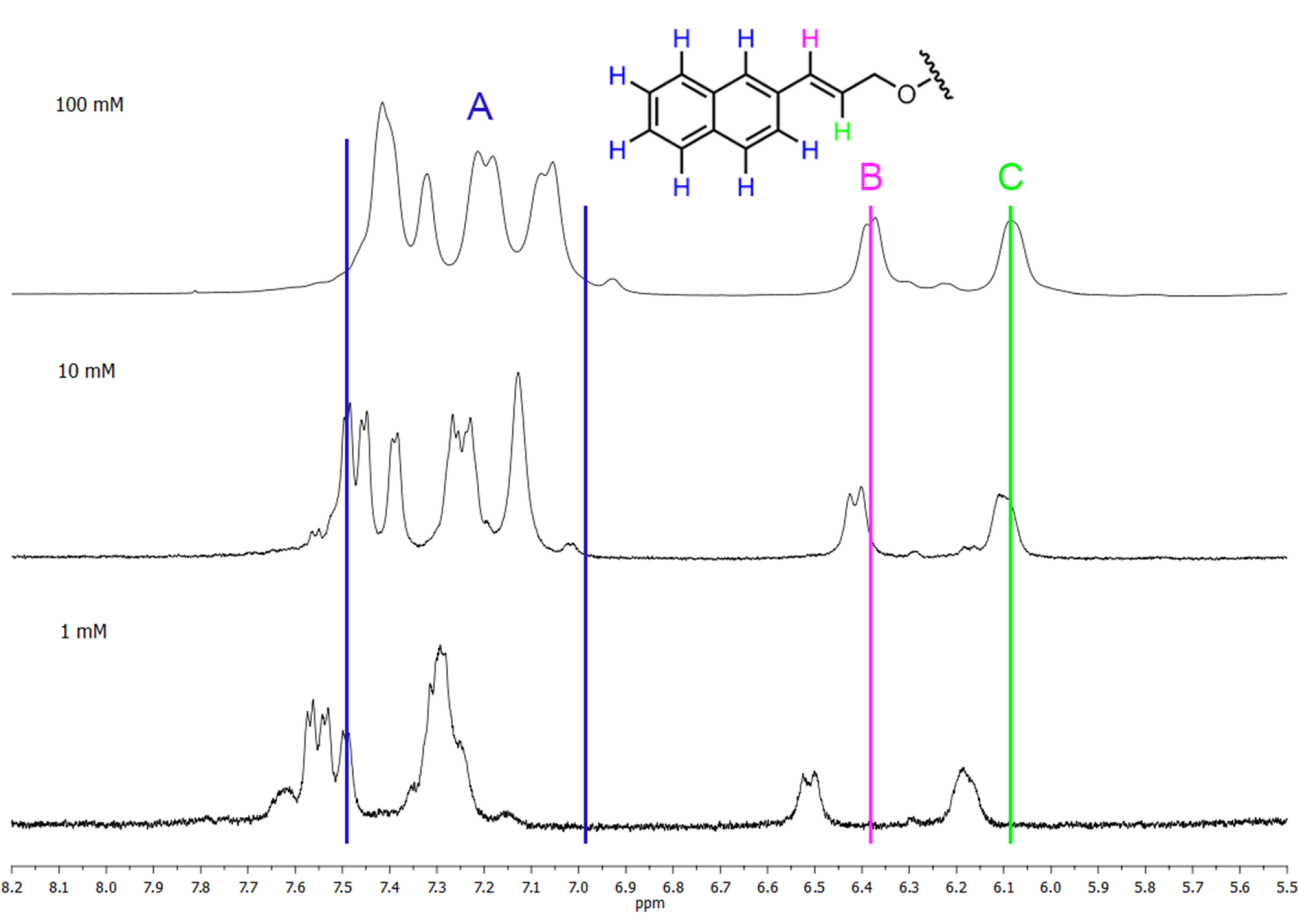
^1^H NMR spectra of 2-*O*-derivative **2a** in D_2_O at concentrations of 100, 10, and 1 mM.

For the 2-*O*-derivative, chemical shifts of hydrogens of the aromatic region and hydrogens of the double bond moved upfield with increasing concentration; therefore, their values are lower (Figure 8). The change in the signal shapes, especially of aromatic hydrogens (A), is also evident. With increasing concentration, the distance between signals B and C of the double bond hydrogens is reduced (about 0.05 ppm), as well as the extent of the area of aromatic hydrogens A (0.13 ppm).

**Figure 9:**
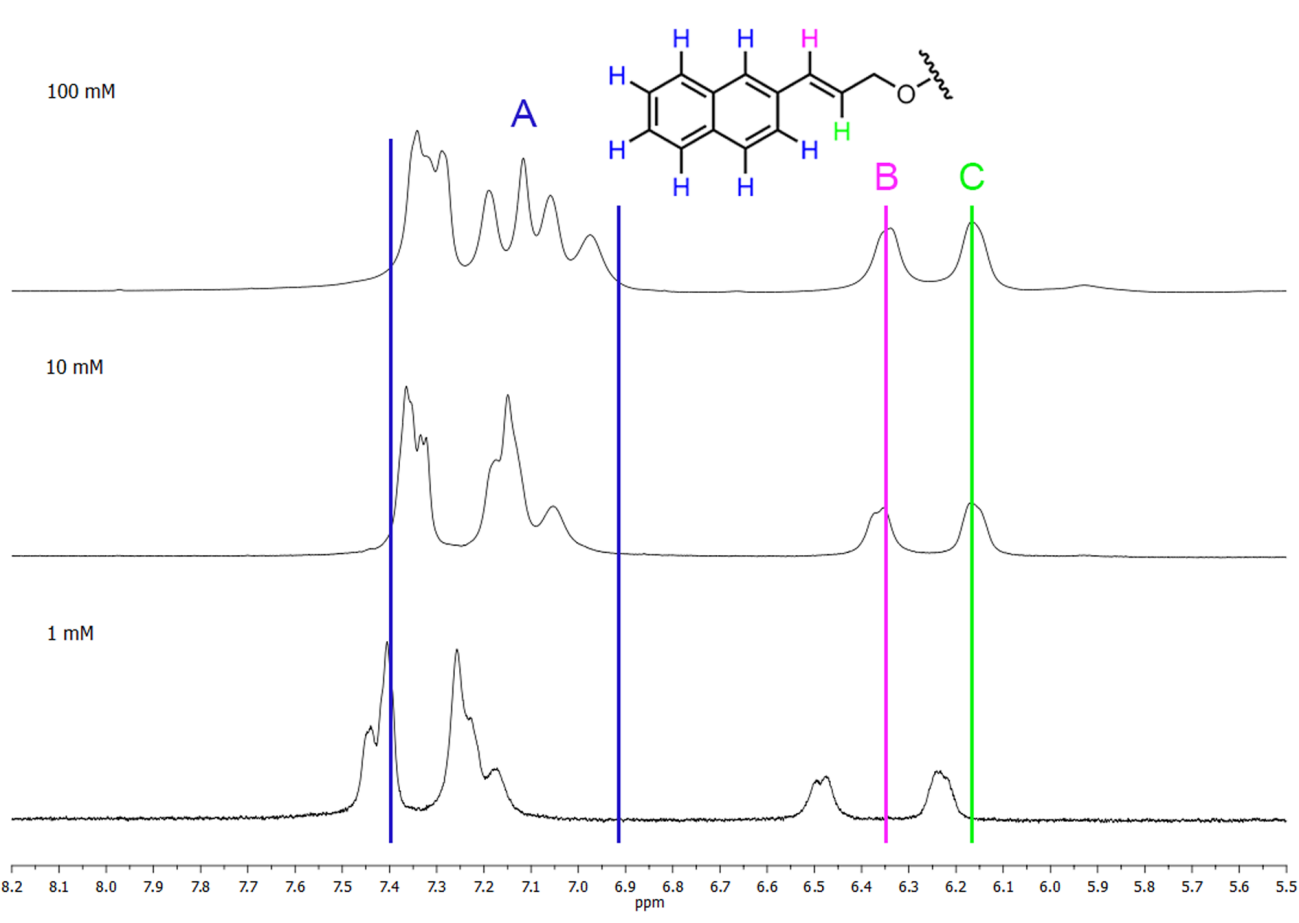
^1^H NMR spectra of 3-*O*-derivative **2b** in D_2_O at concentrations of 100, 10, and 1 mM.

Chemical shifts of hydrogen signals of 3-*O*-derivative at concentrations 100 and 10 mM do not significantly differ (Figure 9). However, a change in the shape of signals and the narrowing (0.12 ppm) of the signals are apparent in the aromatic region (A). For the 1 mM sample, chemical shifts in the aromatic and double bond areas evidently move downfield; however, shapes of peaks do not much differ from the 10 mM sample. It is also worth noting, that while the distance between signals B and C increased (by about 0.07 ppm), the area of aromatic protons A narrowed (about 0.21 ppm).

For all of the above cases, the changes of chemical shifts and shapes of signals in the CD hydrogens’ area were minimal. Also, NOESY and ROESY experiments did not indicate any interaction between the NA group and the CD cavity (data not shown).

### Summarization and discussion of results of different methods

We have successfully employed two synthetic approaches to 2^I^-*O*-, 3^I^-*O*- a 6^I^-*O*-NA derivatives of γ-CD. The first approach is based on a cross-metathesis reaction between corresponding regioisomers of mono-*O*-allyl derivatives of γ-CD and 2-vinylnaphthalene yielding 16–25%; however, the overall yield from γ-CD is only 2–5%. The second approach is based on the direct alkylation of γ-CD, with easy to prepare 3-(naphth-2-yl)allyl alkylation reagent, yielding up to 24%. Moreover, we achieved high regioselectivity in preparations of all regioisomers using the second approach, so this method proved to be superior.

From the NMR experiments, we conclude that the signals of hydrogens of the naphthylallyl group of 2-*O*- and 3-*O*-derivatives differ not only in the shape of peaks, but also in the value of the chemical shifts. For both regioisomers the concentration dependence of chemical shifts in aromatic and allyl areas indicates intermolecular association such as inclusion of the naphthylallyl moiety into the cavity of γ-CD or π stacking of naphthyls. The absence of the significant concentration dependence of chemical shifts of the CD hydrogens does not disqualify inclusion complexation because changes of chemical shifts by 0.01 ppm or less were observed upon inclusion of an aromatic guest into the γ-CD cavity [41]. To reconcile the absence of interactions CD/NA in NOESY and ROESY experiments with NA inclusion into CD is more difficult. Nevertheless, the change in chemical shifts was observed also for the allyl hydrogens which indicates that the allyl group might be also inserted into the CD cavity which would increase the distance between CD and NA hydrogens. Thus, the co-existence of various interactions such as inclusion of NA to various depths of the CD cavity, stacking of naphthyl rings either inside or outside the CD cavity (possibly leading to micellization or aggregation) must be expected.

The intermolecular interactions were quantified by ITC. The obtained values of binding constants were in the upper range of values usually found for the inclusion complexation of γ-CD [42] and decreased in the order (i) 2-*O*-, (ii) 3-*O*-, and (iii) 6-*O*-, as well as with temperature. The values were obtained from the fit to the model in which NA-γ-CD is taken as a ditopic monomer of the AB type with groups A and B undergoing an association of 1:1. However, a number of alternate or competing interactions may occur, as mentioned above, γ-CD is known to form also inclusion complexes of 2:1 stoichiometry and formation of cyclic supramolecular structures my also occur. Moreover to make the situation more complex, the change of the stoichiometry from 1:1 to 2:2 upon the increase in concentration has been reported for a naphthalene–β-CD complex; the concurrently observed increase in excimer indicated close pairing of naphthalene molecules [43]. Nevertheless, the good fit, observed at least for the 2-*O*- and 3-*O*-regioisomers, identifies the 1:1 complexation as the probably dominant associative process.

**Figure 10:**
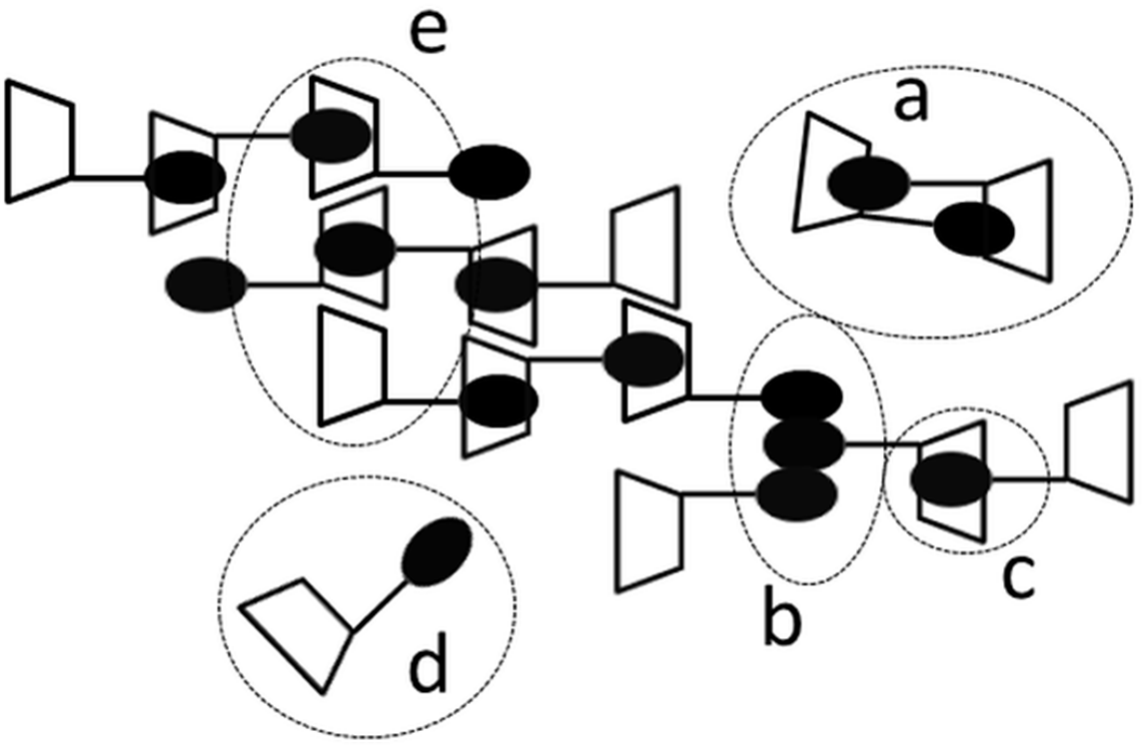
Putative objects and interactions in naphthylallyl-γ-CD solution, depicted schematically for 6^I^-*O*-naphthylallyl-γ-CD (**2c**). (a) Cyclic supramolecular dimer based on inclusion complexation. (b) Interactions between naphthyl groups (stacking) leading to micellization or aggregation. (c) Inclusion complexation of naphthyl into the CD cavity leading to supramolecular polymerization. (d) Free **2c**. (e) Interaction between CD moieties leading to aggregation or precipitation.

The presence of unimers and/or short oligomers was confirmed by DLS even though objects with corresponding hydrodynamic diameters of a few nm were observed only in solutions of the 2-*O*- and 3-*O*-regioisomers after filtration through a filter with 0.22 μm pores. Indeed, larger particles with diameter in the range of tens and hundreds nm dominate the scattering, and such large objects of various morphologies were also detected by cryo-TEM. In this respect, the behavior of NA-γ-CD regioisomers is similar to native CDs for which unstable large objects are also observed [22]. Thus, the supramolecular behavior of NA-γ-CD seems to be determined not only by the interactions of NA and γ-CD but also by interactions of the subparts of the same type, i.e., NA/NA and γ-CD/γ-CD. In Figure 10 various putative interactions of NA-γ-CD are schematically depicted indicating how small supramolecular structures such as supramolecular oligomers or micelles as well as large one such as aggregates can originate.

As all three NA-γ-CD regioisomers were prepared, the effect of the substituent position could be assessed. Particularly, different behavior was observed especially between the 2-*O*-, 3-*O*-regioisomers on the one hand and the 6-*O*-one on the other. There are two underlying structural differences: (i) NA groups face the wider CD side in the head-tail arrangement of 6-*O*-isomers, (ii) the spacer of the 6-*O*-isomer is elongated by a CH_2_ group of glucose unit. The details of differences in behavior in water could not be fully assessed due to the low solubility of the 6-*O*-regioisomer. In fact, the low solubility of the 6-*O*-regioisomer is the most striking difference in behavior of investigated regioisomers. The difference is easier to explain considering inclusion complexation of NA-γ-CD rather than interaction of NA groups only. Consequently, even though no direct evidence for inclusion complexation of NA-γ-CD was obtained it should not be omitted from considerations, especially because the inclusion complexation of naphthalene derivatives with CDs (including γ-CD) is so well established [42].

The experiments performed clearly demonstrate the supramolecular behavior of newly synthesized NA-γ-CDs in solution; at the same time, the experiments showed the anticipated formation of linear NA-γ-CD suprapolymers based on inclusion complexation as a somewhat naive concept – other interactions, e.g., naphthalene stacking may occur between two NA-γ-CD molecules; in addition, mutual interactions of more than two NA-γ-CD molecules may occur, leading to branched supramolecular structures or aggregation. It is to be hoped that the future research not only reveals details of NA-γ-CD interactions but also show how to utilize them in construction of functional supramolecular assemblies.

## Conclusion

New methods for highly regioselective mononaphthylallylation of γ-CD to positions 2-*O*-, 3-*O*-, and 6-*O*- were developed. The alkylation reagent 2-(3-chloroprop-1-enyl)naphthalene, used for the reactions, can be easily prepared in large quantities without the need for chromatographic separation. The method originally expected to be used for the preparation of the regioisomers based on the metathesis of allyl-γ-CD derivatives proved to be inferior, giving about a 6 times lower overall yield than the mononaphthylallylation procedure. Compared to allyl derivatives, the NA-γ-CD derivatives are easy to prepare and purify. Due to the presence of the double bond in the NA group, they can be used as starting compounds for preparation of other monosubstituted γ-CD derivatives.

We demonstrated that the supramolecular properties of individual NA-γ-CD regioisomers substantially differ. The largest difference is being observed between 2-*O*- and 3-*O*-isomers on one hand and the 6-*O*- on the other. To explain the differences in the size of objects observed by different methods, various types of interactions have to be taken into account.

## Supporting Information

File 1 Experimental part and data for compounds **2a**, **2b**, and **2c**: copies of NMR spectra and DLS.
