# Supplementary material for: Synthesis and supramolecular properties of regioisomers of mononaphthylallyl derivatives of γ-cyclodextrin
Source: Beilstein J Org Chem. 2017 Nov 27;13:2509–20. doi: 10.3762/bjoc.13.248 (PMC5727769; doi:10.3762/bjoc.13.248)
Supplement: File 1 — Experimental part and data for compounds 2a, 2b, and 2c: copies of NMR spectra and DLS. [file Beilstein_J_Org_Chem-13-2509-s001.pdf]

Supporting Information

for

Synthesis and supramolecular properties of regioisomers of mononaphthylallyl derivatives of γ-cyclodextrin

Markéta Bláhová[1], Sergey K. Filippov[2], Lubomír Kováčik[3], Jiří Horský[2], Simona Hybelbauerová[4], Zdenka Syrová[3], Tomáš Křížek[5] and Jindřich Jindřich[1]*

Address: [1]Department of Organic Chemistry, Faculty of Science, Charles University, Hlavova 8, 128 43 Prague 2, Czech Republic, [2]Institute of Macromolecular Chemistry, Academy of Sciences of the Czech Republic, Heyrovsky Sq. 2, 162 06 Prague 6, Czech Republic, [3]Institute of Biology and Medical Genetics, First Faculty of Medicine, Charles University, Albertov 4, 128 01 Prague 2, Czech Republic, [4]Department of Teaching and Didactics of Chemistry, Faculty of Science, Charles University, Hlavova 8, 128 43, Prague 2, Czech Republic and [5]Department of Analytical Chemistry, Faculty of Science, Charles University, Hlavova 8, 128 43, Prague 2, Czech Republic

Email: Jindřich Jindřich - jindrich@natur.cuni.cz

*Corresponding author

Experimental part and data for compounds 2a, 2b, and 2c: copies of NMR spectra and DLS

Contents

Experimental part S4

General information S4

Synthesis S5

Per-*O*-acetyl-2^{I}-*O*-allyl-γ-cyclodextrin (**1a**), per-*O*-acetyl-3^{I}-*O*-allyl-γ-cyclodextrin (**1b**), per-*O*-acetyl-6^{I}-*O*-allyl-γ-cyclodextrin (**1c**) S5

2^{I}-*O*-(3-(Naphthalen-2-yl) prop-2-en-1-yl-γ-cyclodextrin (**2a**) S5

3^{I}-*O*-(3-(Naphthalen-2-yl) prop-2-en-1-yl-γ-cyclodextrin (**2b**) S7

6^{I}-*O*-(3-(Naphthalen-2-yl) prop-2-en-1-yl-γ-cyclodextrin (**2c**) S8

Naphthalene-2-carbaldehyde (**3**) S9

Ethyl-3-(naphthalen-2-yl) acrylate (**4**) S10

Ethyl malonic half ester (**5**) S10

3-(Naphthalen-2-yl) prop-2-en-1-ol (6), 2-(3-chloroprop-1-enyl)naphthalen (**7**) S10

DLS S10

Cryo-TEM S11

ITC S12

NMR spectra of 2^{I}-*O*-(3-(naphthalen-2-yl)prop-2-en-1-yl-γ-cyclodextrin (**2a**) S15

^{1}H **2a** S15

^{13}C **2a** S15

^{1}H-^{1}H – COSY **2a** S16

HSQC **2a** S17

HMBC 2a S17

NMR spectra of 3^I^-*O*-(3-(naphthalen-2-yl)prop-2-en-1-yl-γ-cyclodextrin (**2b**) S18
^{1}H **2b** S18
^{13}C **2b** S18
^{1}H-^{1}H – COSY **2b** S19
HSQC **2b** S20
HMBC **2b** S20
NMR spectra of 6^I^-*O*-(3-(naphthalen-2-yl)prop-2-en-1-yl-γ-cyclodextrin (**2c**) S21
^{1}H **2c** S21
^{13}C **2c** S21
^{1}H-^{1}H – COSY **2c** S22
HSQC **2c** S23
HMBC **2c** S23
DLS S24
Correlation functions S24
Scattering intensity S25
γ-CD solution behavior S25
References S27

Experimental part

General information

γ-CD was purchased from Wako Chemicals (Germany), special chemicals and solvents from Sigma-Aldrich and common solvents from Penta or Lach-ner (Czech Republic). γ-CD was dried at 70 °C under reduced pressure (3 mbar). Solvents were distilled before use. DMSO was distilled and dried with 3 Å molecular sieves. DMF was predried with P_2O_5, then distilled and dried with 3 Å molecular sieves. Redistilled water was used.

EtONa was prepared by dissolving of Na (1.8 g) in absolute EtOH (30 ml). Residual solvent was then evaporated to yield solid EtONa. NaH was used as a 60% suspension in mineral oil. LDA was used as a 1.5 M solution in cyclohexane, in complex with THF in ratio 1:1.

Cation exchanger DOWEX 50 was activated and converted to its H^+ form using standard methods and finally washed with 50% aqueous MeOH. Silica gel 60 (0.040–0.063 mm, Merck, Germany) was used for chromatography. TLC was performed on silica gel 60 F_{254}-coated aluminum sheets (Merck, Germany) or reverse silica gel 60 RP-18 F_{254}S (Merck, Germany). Spots were detected by a UV lamp (λ = 254 nm) or by spraying with 50% aqueous H_2SO_4 followed by carbonization with a heat-gun.

NMR spectra were recorded on a Bruker Avance III (600 MHz) (^{1}H at 600.17 MHz, ^{13}C NMR at 150.04 MHz) and on a Varian VNMRS 300 (^{1}H at 299.94 MHz) in deuterated solvents and are referenced to the residual solvent peak. Chemical shifts are given in δ-scale, coupling constants *J* are given in Hz. The numbering of atoms for NMR spectra transcription was done analogous to **Figure S1:** Numbering of atomsFigure S1. The glucose unit bearing an alkyl substituent is numbered with “^{I}” sign where the assignment is unambiguous. All other glucose signals are numbered indiscriminately. The alkyl substituent is numbered with “′”

sign. Label 4’ is used for all aromatic hydrogens of the naphthyl substituent. The assignment of the ^{1}H and ^{13}C signals was based on 2D NMR techniques (^{1}H-^{1}H COSY, HSQC, HMBC).

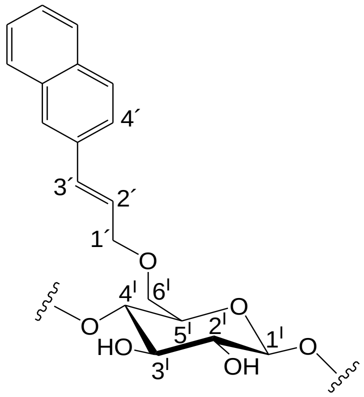

Figure S1: *Numbering of atoms*

Ozonolysis was performed in an Ozone Tech Systems (Sweden) ACT-3000 apparatus.

Mass spectra MS-ESI were measured on an Esquire 3000 (Bruker).

Melting points (uncorrected) were determined using a Kofler apparatus. Specific rotations ($[\alpha]^{20}_{D}$) were measured in concentration around 10 g L^{-1} at 20 °C. Infrared spectra were recorded in KBr, measured on a Hermo Nicolet AVATAR 370 FT-IR spectrometer, and are reported in wave numbers (cm^{-1}). HRMS (MALDI) spectra were recorded on an Agilent Technologies 6530 Accurate-Mass Q-TOF LC/MS.

Synthesis

Per-*O*-acetyl-2^I^-*O*-allyl-γ-cyclodextrin (1a), per-*O*-acetyl-3^I^-*O*-allyl-γ-cyclodextrin (1b), per-*O*-acetyl-6^I^-*O*-allyl-γ-cyclodextrin (1c)

Preparation is described in our previous publication [1].

2^I^-*O*-(3-(Naphthalen-2-yl) prop-2-en-1-yl-γ-cyclodextrin (2a)

Method A: Per-*O*-acetyl-2^I^-*O*-allyl-γ-cyclodextrin (**1a**, 200 mg, 0.087 mmol) was dissolved in benzene (1 ml). Subsequently, 2-vinylnaphthalene (13 mg, 0.087 mmol) and Hoveyda–Grubbs 2nd generation catalyst (5 mg, 0.009 mmol) were added. The mixture was stirred

overnight at 75 °C. The reaction was monitored by TLC ($CHCl_3$/MeOH, 20/1). The solvent was then evaporated and products were separated by chromatography on a silica gel column ($CHCl_3$/MeOH, gradient from 70/1 to 50/1). The product obtained in this way still contained some starting material. The mixture (177 mg) was then deacetylated by adding NaOMe in MeOH (0.1 M, 4 ml) and stirred overnight; the reaction mixture turned milky white. The reaction was monitored by TLC (PrOH/H_2O/EtOAc/conc. aq. NH_3, 6/3/1/1). There was a spot of γ-CD present on TLC as well because of the decomposition of the starting material by the catalyst. The reaction mixture was dissolved in water (10 ml), DOWEX 50 in H^+ form (1 g) was added, and the mixture was stirred for 0.5 h. DOWEX was then filtered off on a medium sized frit (S2) and washed with 50% MeOH. Solvents were evaporated from the filtrate, dissolved in water. The above described procedure using DOWEX was repeated once more. The mixture of the product, starting material and γ-CD (100 mg) was adsorbed on silica gel (0.5 g). The product was separated by chromatography on silica gel column using the elution mixture (PrOH/H_2O/conc. aq. NH_3, 8/3/1). The main product **2a** was isolated in the form of a white solid in 25% yield (32 mg, overall yield based on γ-CD 5%).

Method B: Dry γ-CD (1.75g, 1.35 mmol) was dissolved in dry DMSO (10 ml) under argon atmosphere, then NaOH was added (540 mg, 13.50 mmol). The reaction mixture was stirred overnight at 20 °C. Then, 2-(3-chloroprop-1-enyl) naphthalene (275 mg, 1.35 mmol) was added and the reaction mixture was stirred overnight. The reaction was monitored by TLC (PrOH/H_2O/EtOAc/conc. aq. NH_3, 6/3/1/1). The reaction was stopped by neutralization with 50% H_2SO_4 and the products precipitated in acetone (350 ml). The precipitate was filtered through medium sized frit (S3) and washed with acetone. The air-dried precipitate was then dissolved in hot water (50 ml) and silica gel (10 g) was added. The mixture was evaporated and the products adsorbed on silica gel were separated by chromatography on silica gel

column (($PrOH/H_2O$)/conc. aq. NH_3, 8/3/1). The yield of the main product **2a** was 14% (270 mg).

^{1}H NMR (600 MHz, DMSO-d_6): δ = 7.94–7.83 (m, 4 H, 4 × H-4´), 7.73–7.69 (m, 1 H, 1 × H-4´), 7.55–7.43 (m, 2 H, 2 × H-4´), 6.84 (d, J = 15.9 Hz, 1 H, H-3´), 6.50 (dt, J = 16.0, 6.2 Hz, 1 H, H-2´), 5.98 (d, J = 2.7 Hz, 1 H, 1 × OH-3^I), 5.92–5.61 (m, 13H, 13 × OH), 5.16 (d, J = 3.8 Hz, 1 H, 1 × H-1^I), 4.93–4.78 (m, 7 H, 7 × H-1), 4.62–4.40 (m, 11H, 9 × OH), 4.48 (2 H, 2 × H-1´), 3.78 (dd, J = 9.2, 9.2 Hz, 1 H, 1 × H-3^I), 3.69–3.20 (m, 82 H, 8 × H-2, 7 × H-3, 8 × H-4, 8 × H-5, 16 × H-6, overlap with H_2O signal) ppm. ^{13}C NMR (151 MHz, DMSO-d_6): δ = 134.43 (C-4´), 133.64 (C-4´), 133.08 (C-4´), 132.77 (C-3´), 128.68 (C-4´), 128.40 (C-4´), 128.06(C-4´), 127.26 (C-2´), 126.93 (C-4´), 126.76 (C-4´), 126.56 (C-4´), 124.18 (C-4´), 102.42 –101.91 (6 × C-1), 101.74 (C-1), 100.24 (C-1^I), 81.70 – 80.83 (8 × C-4), 80.01 (C-2^I), 73.90–72.21 (7 × C-2, 8 × C-3, 8 × C-5), 72.20 (C-1´), 60.74–60.16 (8 × C-6) ppm. IR (drift KBr): ν = 1369, 1155, 1080, 1028 cm^{-1}. HRMS(MALDI): m/z calcd. for $C_{61}H_{90}O_{40}Na$ $[M+Na]^+$ 1485.4906; found 1485.4885. R_f ($PrOH/H_2O/EtOAc/NH_3$, 6/3/1/1) = 0.39. T_{dec} = 280 °C. $[\alpha]^{20}_D$ +120.3° (c 1.00, DMSO).

3^I-*O*-(3-(Naphthalen-2-yl) prop-2-en-1-yl-γ-cyclodextrin (2b)

Method A: **2b** was prepared from the per-*O*-acetyl-3^I-*O*-allyl-γ-cyclodextrin (**1b**, 322 mg, 0.140 mmol), 2-vinylnaphthalene (22 mg, 0.140 mmol) and Hoveyda–Grubbs 2nd generation catalyst (9 mg, 0.014 mmol) were dissolved in benzene (1.6 ml). The previously described procedure (same as for **2a**) afforded 40 mg of the main product **2b** (19% yield, overall yield based on γ-CD 2%) as a white solid.

Method B: Dry γ-CD (1.75g , 1.35 mmol), EtONa (2.30g, 1.35 mmol) as a base and 2-(3-chloroprop-1-enyl)naphthalene (334 mg, 1.35 mmol) as an alkylation agent were mixed

together in DMSO. Previously described procedure (same as for **2a**) afforded 378 mg (19%) of the main product **2b**.

^{1}H NMR (600 MHz, DMSO-d_6): δ = 7.93–7.85 (m, 4 H, 4 × H-4´), 7.75–7.72 (m, 1 H, 1 × H-4´), 7.59–7.52 (m, 2 H, 2 × H-4´), 6.83 (d, J = 15.8 Hz, 1 H, H-3´), 6.54 (dt, J = 15.9, 6.7 Hz, 1 H, H-2´), 6.00 (bs, 1H, 1 × OH-2^{I}), 5.90–5.69 (m, 13 H, 13 × OH), 5.00 (d, J = 3.6 Hz,1 H,1 × H-1), 4.96 – 4.86 (m, 7 H, 7 × H-1), 4.64–4.44 (m, 11 H, 9 × OH), 4.58 (2 H, 2 × H-1´), 3.80 – 3.22 (m, 54 H,7 × H-2, 7 × H-3, 7 × H-4, 8 × H-5, 16 × H-6, overlap with H_2O signal), 3.72 (1 H, 1 × H-3^{I}), 3.57 (1 H, 1 × H-4^{I}), 3.28 (1H, 1 × H-2^{I}) ppm. ^{13}C NMR (151 MHz, DMSO-d_6): δ = 134.49 (C-4´), 134.26 (C-3´), 133.66 (C-4´), 133.11 (C-4´), 128.70 (C-4´), 128.34 (C-4´), 127.96 (C-4´), 127.26 – 126.88 (2 × C-4´), 126.54 (C-2´), 126.67 (C-4´), 123.84 (C-4´), 102.46 –101.86 (7 × C-1), 100.94 (C-1), 81.84 – 81.56 (3 × C-4), 81.39 (2 × C-4), 81.06 (C-4), 80.92 (C-4), 79.45 (C-3), 76.94 (C-4), 74.32–72.50 (7 × C-2, 7 × C-3, 8 × C-5), 73.84 (C-2), 73.11 (C-1´), 60.48–59.66 (8 × C-6) ppm. IR (drift KBr): ν = 1370, 1155, 1080, 1028 cm^{-1}. HRMS(MALDI): m/z calcd. for $C_{61}H_{90}O_{40}Na$ [M+Na]$^{+}$ 1485,4906; found 1485.4881. R_f (PrOH/H_2O/EtOAc/NH_3, 6/3/1/1) = 0.33. t_{dec} = 281 °C. $[\alpha]^{20}_{D}$ + 93.6° (c 1.00, DMSO).

6^{I}-*O*-(3-(Naphthalen-2-yl) prop-2-en-1-yl-γ-cyclodextrin (2c)

Method A: **2c** was prepared from the per-*O*-acetyl-6^{I}-*O*-allyl-γ-cyclodextrin (**1c**, 48 mg, 0.021 mmol), 2-vinylnaphthalene (3 mg, 0.021 mmol) and Hoveyda–Grubbs 2nd generation catalyst (1 mg, 0.002 mmol) were dissolved in benzene (0.3 ml). The previously described procedure (same as for **2a**) afforded 5 mg of **2c** as the main product (16% yield, overall yield based on γ-CD 3%) as a white solid.

Method B: Dry γ-CD (1.10g, 0.84 mmol), NaH (405 mg, 10.13 mmol) as a base and 2-(3-chloroprop-1-enyl)naphthalene (171 mg, 0.84 mmol) as an alkylation agent were mixed

together in DMSO. Previously described procedure (same as for **2a**) afforded 155 mg (12%) of the main product **2c**.

^{1}H NMR (600 MHz, DMSO-d_6): δ = 7.88 – 7.85 (m, 4H, 4 × H-4´), 7.73 – 7.71 (m, 1 H, H-4´), 7.53 – 7.47 (m, 2 H, 2 × H-4´), 6.76 (d, *J* = 15.8 Hz, 1H, H-3’), 6.55 – 6.51 (m, 1H, H-2’), 5.83 (bs, 16H, 8 × OH-2, 8 × OH-3), 4.91 (m, 8H, 8 × H-1), 4.70 – 4.44 (m, 7H, 7 × OH-6), 4.27 – 4.23 (m, 1H, H-1’), 4.17 – 4.14 (m, 1H, H-1’), 3.76 – 3.32 (m, 48H, 8 × H-2, 8 × H-3, 8 × H-4, 8 × H-5, 16 × H-6, overlap with H_2O signal) ppm. ^{13}C NMR (125 MHz, DMSO-d_6) δ = 134.12 (C-4´), 133.22 (C-4´), 132.51 (C-4´), 130.94 (C-3’), 128.11 (C-4´), 127.97 (C-2’), 127.82 (C-4´), 127.71 (C-4´), 127.53 (C-4´), 126.49 (C-4´), 125.94 (C-4´), 123.72 (C-4´), 102.32 (C-1^{I}), 101.76 – 101.70 (m, 7 × C-1), 81.88 (C-4), 81.29 (C-4), 81.04 – 80.87 (6 × C-4), 72.91 – 72.12 (m, 8 × C-2, 8 × C-3, 7 × C-5), 71.25 (C-1’), 70.81 (C-5^{I}), 68.95 (C-6^{I}), 60.24 – 59.92 (7 × C-6) ppm. IR (drift KBr): ν = 1371, 1155, 1079, 1028 cm^{-1}. HRMS(MALDI): m/z calcd. for $C_{61}H_{90}O_{40}Na$ $[M+Na]^+$ 1485.4906; found 1485.4833. R_f (PrOH/H_2O/EtOAc/NH_3, 6/3/1/1) = 0.28. t_{dec} = 278 °C. $[\alpha]^{20}_{D}$ +116.7° (*c* 1.00, DMSO).

Naphthalene-2-carbaldehyde (3)

2-Vinylnaphthalene (800 mg, 5.188 mmol) was dissolved in CH_2Cl_2:MeOH (5:1, 6 ml) and ozonolyzed at –78 °C for 3 h. The reaction was monitored with TLC (hexane/toluene, 1/1). The reaction mixture was quenched by adding Me_2S (2 ml) and the solvents were evaporated. Purification of the crude product using column chromatography on silica gel ($CHCl_3$/MeOH, 70/1) yielded 590 mg (73%) of **3** as a white solid.

^{1}H NMR (300 MHz, $CDCl_3$): δ = 10.17 (s, 1 H, CHO), 8.42 – 7.49 (m, 7 H, 7 × H-Ar) ppm. [2]

Ethyl-3-(naphthalen-2-yl) acrylate (4)

Naphthalene-2-carbaldehyde (**3**, 914 mg, 5.85 mmol) was dissolved in pyridine (7.7 ml), then monoethylmalonate (1.68 g, 11.41 mmol) and piperidine (0.15 ml) were added. The mixture was heated to 120 °C for 6 h. The reaction was monitored with TLC (hexane/EtOAc, 2/1). Pyridine was evaporated, the residue was dissolved in EtOAc (20 ml) and washed with $NaHCO_3$ (5% solution, 3 × 50 ml). The organic phase was dried with $MgSO_4$. The product was adsorbed on silica gel (9 g) and the purification by column chromatography (hexane/EtOAc, 20/1) yielded 1.072 g (81%) of compound **4** as a white solid.

^{1}H NMR (300 MHz, $CDCl_3$): δ = 8.00 – 7.41 (m, 8 H, 7 × H-Ar, 1 × CH), 6.56 (d, J = 16.0 Hz, 1 H, 1 × CH), 4.30 (q, J = 7.1 Hz, 2 H, 1 × CH_2), 1.37 (t, J = 7.1 Hz, 3 H, 1 × CH_3) ppm. [3]

Ethyl malonic half ester (5)

Compound **5** was prepared according to the known procedure [4].

3-(Naphthalen-2-yl)prop-2-en-1-ol (6), 2-(3-chloroprop-1-enyl)naphthalene (7)

Compounds **6** and **7** were prepared according to the known procedure [3].

DLS

The particles' hydrodynamic radius, R_H, and scattering intensity, I_S, were measured at a scattering angle of θ = 173° using a ZetasizerNano ZS instrument, model ZEN3600 (Malvern Instruments, U.K.). Measurements were repeated from three to five times. Correlation functions $g^2(t)$ were analyzed by a regularized inverse Laplace transformation by the REPES algorithm, which provided distributions $A(\tau)$, of relaxation times τ according to

$$g^2(t) = 1 + \beta\left[\int A(t)\exp(-t/\tau)d\tau\right]^2,$$

where β is an instrumental factor. For the diffusion of nanoparticles in liquid, the hydrodynamic radius R_H or hydrodynamic diameter D_H can be determined using the Stokes–Einstein equation

$$D = (\tau q^2)^{-1},$$

$$q = (4\pi n/\lambda_0)\sin(\vartheta/2)\; D = kT/6\pi\eta R_H,$$

where k is the Boltzmann constant, n the refractive index and η the viscosity of the solvent.

Cryo-TEM

Sample preparation

The 2-*O*- and 3-*O*-NA-γ-CD were used as a 2, 20 and 100 mM solution in water, the 6-*O*-NA-γ-CD as 0.5 mM and 5 mM aqueous solutions. The isomers were dissolved in an appropriate amount of deionized filtrated water and stirred in a closed vial for 10 min at 120 °C.

Measurement

Cryo-TEM measurements were carried out using a Tecnai G^2 Sphera 20 electron microscope (FEI Company, Hillsboro, OR, USA) equipped with a Gatan 626 cryo-specimen holder (Gatan, Pleasanton, CA, USA) and a LaB_6 gun. The samples for cryo-TEM were prepared by plunge-freezing (Dubochet, 1988). Briefly, 3 μL of the sample solution were applied to a copper electron microscopy grid covered with a perforated carbon film forming woven-mesh-like openings of different sizes and shapes (the lacey carbon grids #LC-200 Cu, Electron Microscopy Sciences, Hatfield, PA, USA), glow discharged for 40 s with a 5 mA current. Most of the sample was removed by blotting (Whatman no. 1 filter paper) for approximately 1 s, and the grid was immediately plunged into liquid ethane held at –183 °C. The grid was then transferred without rewarming into the microscope. Images were recorded at an accelerating

voltage of 120 kV and with magnifications ranging from 11500× to 50000× using a Gatan UltraScan 1000 slow scan CCD camera in the low-dose imaging mode, with the electron dose not exceeding 1500 electrons per nm^2. The magnifications resulted in final pixel size ranging from 1 to 0.2 nm, the typical value of applied underfocus ranged between 0.5 to 2.5 μm. The applied blotting conditions resulted in the specimen thickness varying between 100 and ca. 300 nm. Specimens were sonicated for 10 minutes with a Diogenode Bioruptor sonicator (Tosho Denki Co. Ltd., Japan).

Image processing

Acquired cryo-TEM images with pixel sizes of 0.3 nm and smaller were corrected for the contrast reversals caused by oscillations of the TEM's contrast transfer fuction by the GCTF programme [5] and band-pass filtered with ImageJ [6] in order to suppress density variations caused by changes of ice thickness and noise beyond the cutoff of 1 nm. Images with coarser pixel sizes were only band-pass filtered with the same settings. All distances were measured with ImageJ.

ITC

Measurements

Isothermal titration calorimetry was carried out as dilution experiments at 10, 25 and 45 °C, using the ITC-200 instrument (MicroCal, Inc., Northampton, MA). In each titration, a series 0.5μL + 19 × 2 μL of 10 mM sample was injected into the measuring cell, initially filled with pure water, and the released heat was recorded for each injection. However, the dimer-dissociation procedure of MicroCal Origin for ITC supplied with the instrument could not be used for the extraction of thermodynamic parameters from the obtained data because NA-γ-CDs have to be treated as monomers of AB type, capable of polymerization. Moreover, there

has been some controversy about implementation of the MicroCalc procedure [7]. Therefore the data were treated as described in the following section.

Data treatment

The measuring cell of the isothermal calorimeter has a fixed active volume V_0 fully filled with solution in which component X is at concentration $c_{X,i-1}$. Injection of solution from the syringe (concentration of X $c_{X,S}$) drives an equal volume ΔV_i of initial solution from the active volume to the cell stem. After that, a new concentration $c_{X,i}$ is established due to mixing [8].

$$c_{X,i} = (1 - \frac{\Delta V_i}{V_0})c_{X,i-1} + \frac{\Delta V_i}{V_0}c_{X,S} \quad (3)$$

c_X Is a total concentration of X, that is, the sum of concentrations of free and bound X. Isodesmic supramolecular polymerization [9] of A-B monomer [10] can be used as a reference model of NA-γ-CD behavior in aqueous solution. The extent of binding between A and B can be expressed as a 1:1 binding between A and B described by the binding constant K

$$K = \frac{[AB]}{[A][B]} \quad (4)$$

For an A-B monomer $c_A = c_B = c_{A\text{-}B}$ and [A] = [B] = $c_{A\text{-}B}$ – [AB]; A-B denotes the monomer A-B whereas AB denotes the complex of A and B groups. Then the degree of complexation θ is given by

$$\theta = \frac{[AB]}{c_{A\text{-}B}} = 1 + \frac{1}{2Kc_{A\text{-}B}} - \sqrt{(1 + \frac{1}{2Kc_{A\text{-}B}})^2 - 1} \quad (5)$$

The free and bound groups A and B are in equilibrium in the syringe; the concentration of complexed groups $[AB]_S = c_{AB,S}$ can be obtained from Eq. 5. We assume that mechanical and complexation processes associated with each injection can be separated to two consecutive

steps. Hence, Eq. 3 can be used not only for calculation of the total monomer concentration $c_{\text{A-B},i}$ but also for calculation of the complex concentration prior to equilibration, $(c_{\text{AB},i})_\text{p}$. Since the total concentration of the monomer $c_{\text{A-B},i}$ is not affected by equilibration, the final equilibrium concentration of complex $c_{\text{AB},i}$ can be calculated from Eq. 5. The heat released or absorbed per injection due to AB dissociation, NDH_i normalized to 1 mol of added monomer, is then given by

$$NDH_i = \frac{\Delta H V_0}{c_{\text{AB,S}} \Delta V_i} \left(c_{\text{AB},i} - \left(c_{\text{AB},i} \right)_\text{p} \right) \tag{6}$$

where ΔH is a molar binding enthalpy. Using Eqs 3, 4 and 6 values of K and ΔH can be extracted from titration experiment by an appropriate fitting procedure.

Taking naphthyl and cyclodextrin moieties as A and B groups, the above equations were used for extracting thermodynamic parameters for self-complexation of all three regioisomers NA-γ-CD at temperatures 10 °C, 25 °C, and 45 °C. Experimental values of NDH were fitted to Eqs 3, 4, and 6 using procedures implemented in MS Excel 2010 Solver. One or more initial points were excluded from the fitting.

NMR spectra of 2^{I}-*O*-(3-(naphthalen-2-yl)prop-2-en-1-yl-γ-cyclodextrin (2a)

^{1}H 2a

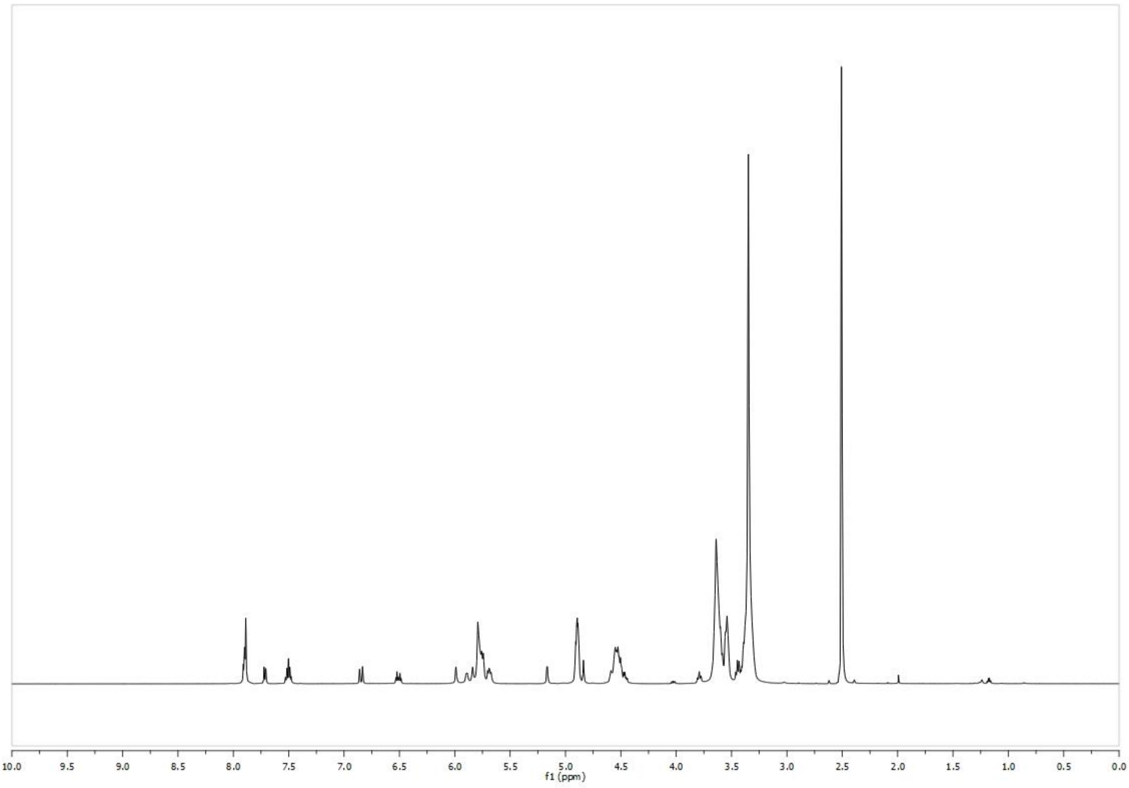

^{13}C 2a

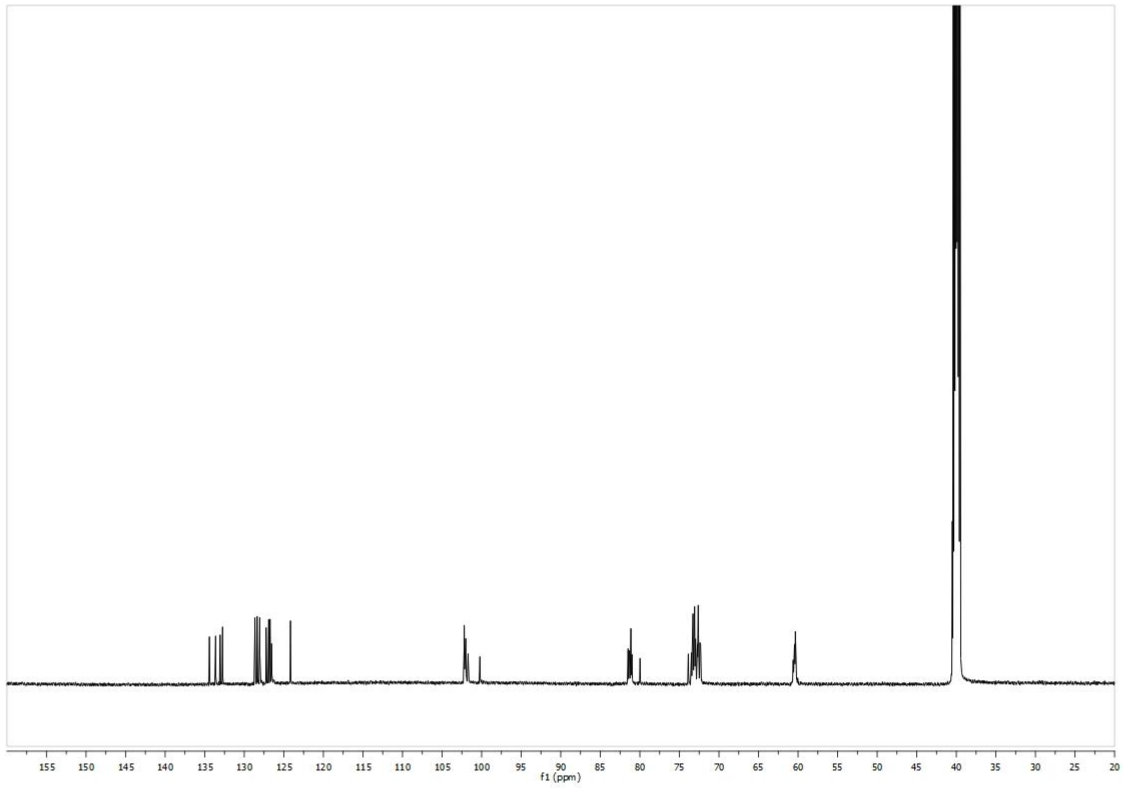

^{1}H-^{1}H – COSY 2a

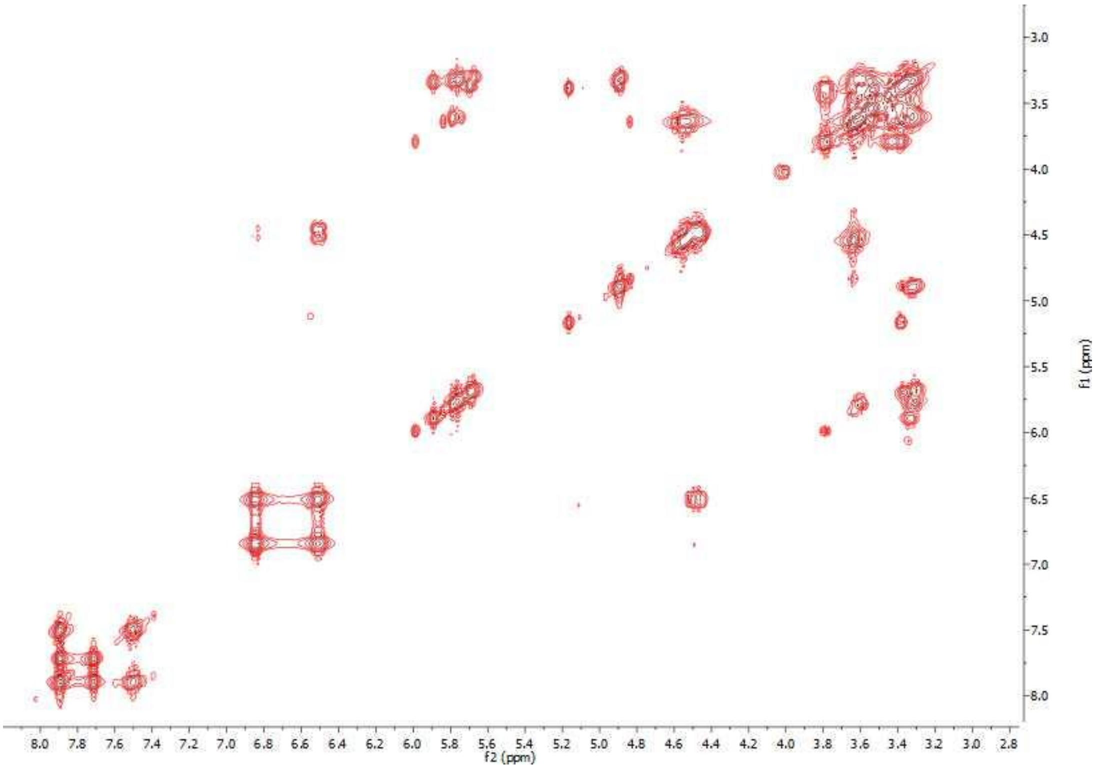

HSQC 2a

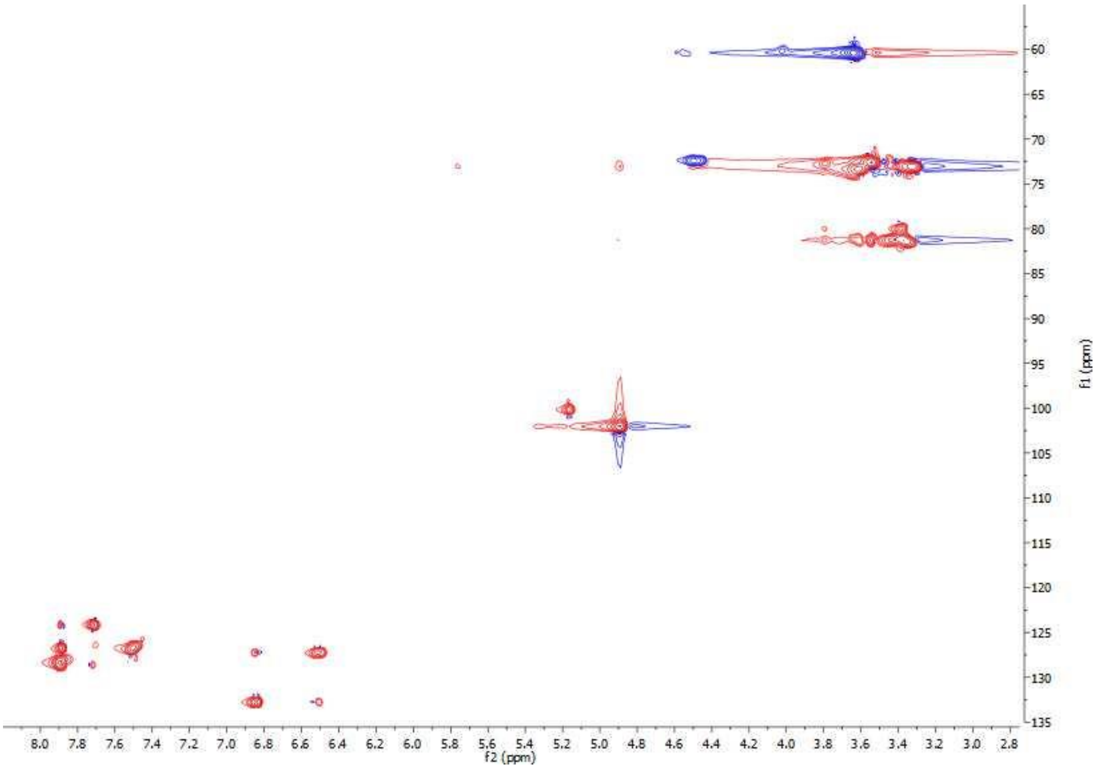

HMBC 2a

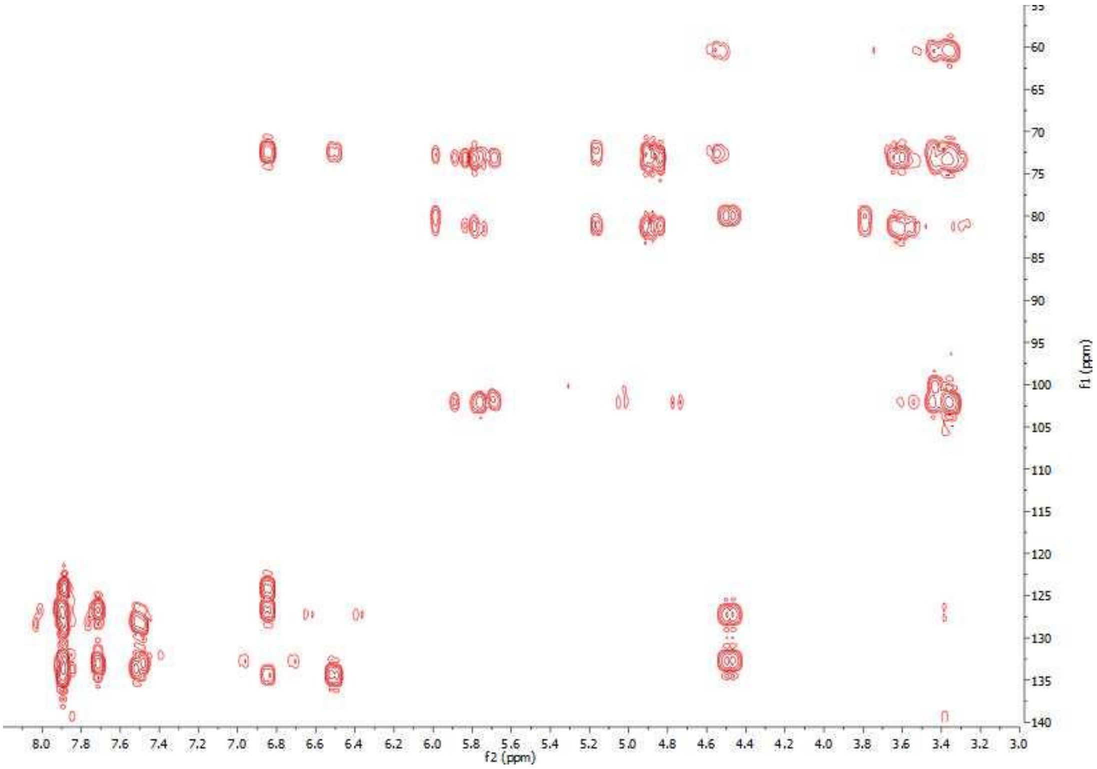

NMR spectra of 3^I^-*O*-(3-(naphthalen-2-yl)prop-2-en-1-yl-γ-cyclodextrin (2b)

^{1}H 2b

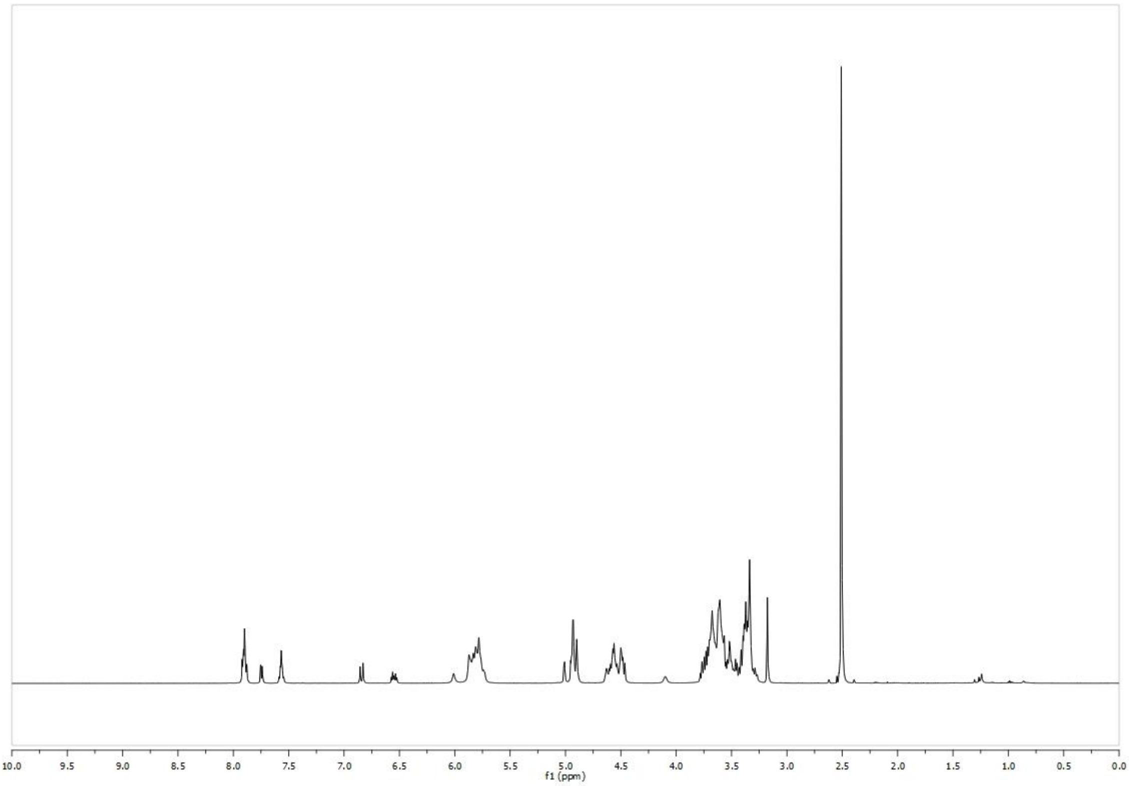

^{13}C 2b

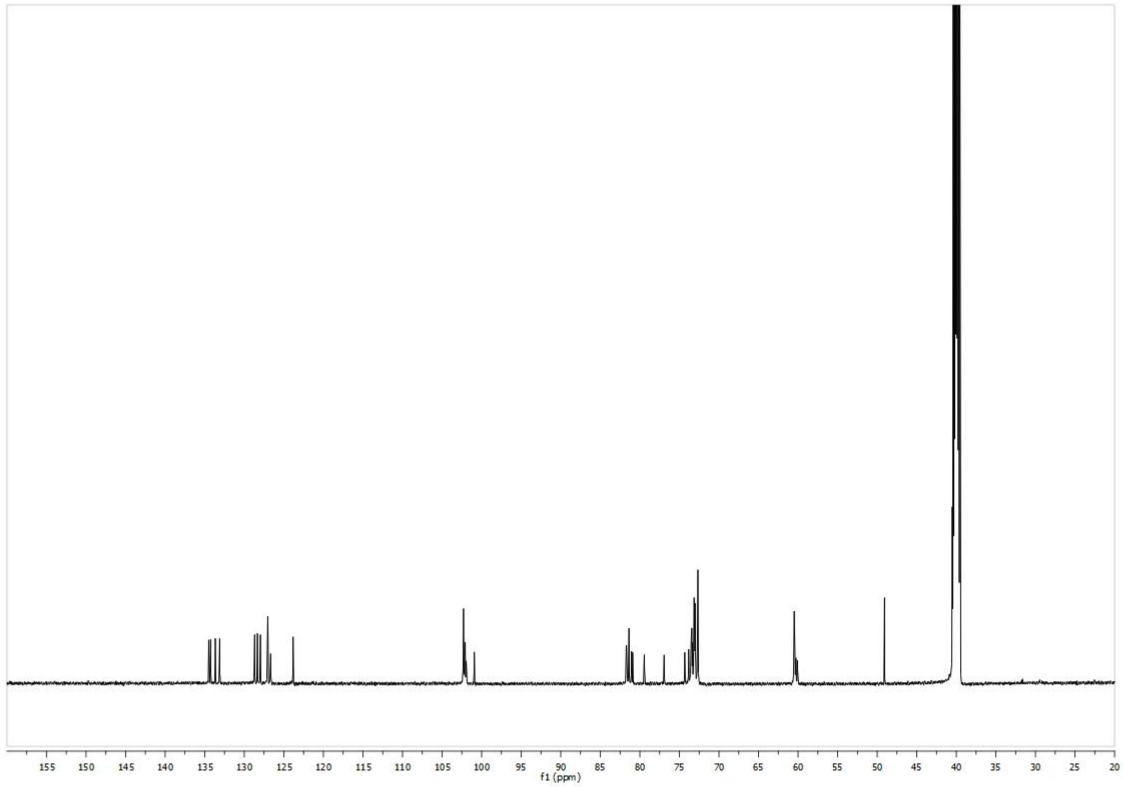

^{1}H-^{1}H – COSY 2b

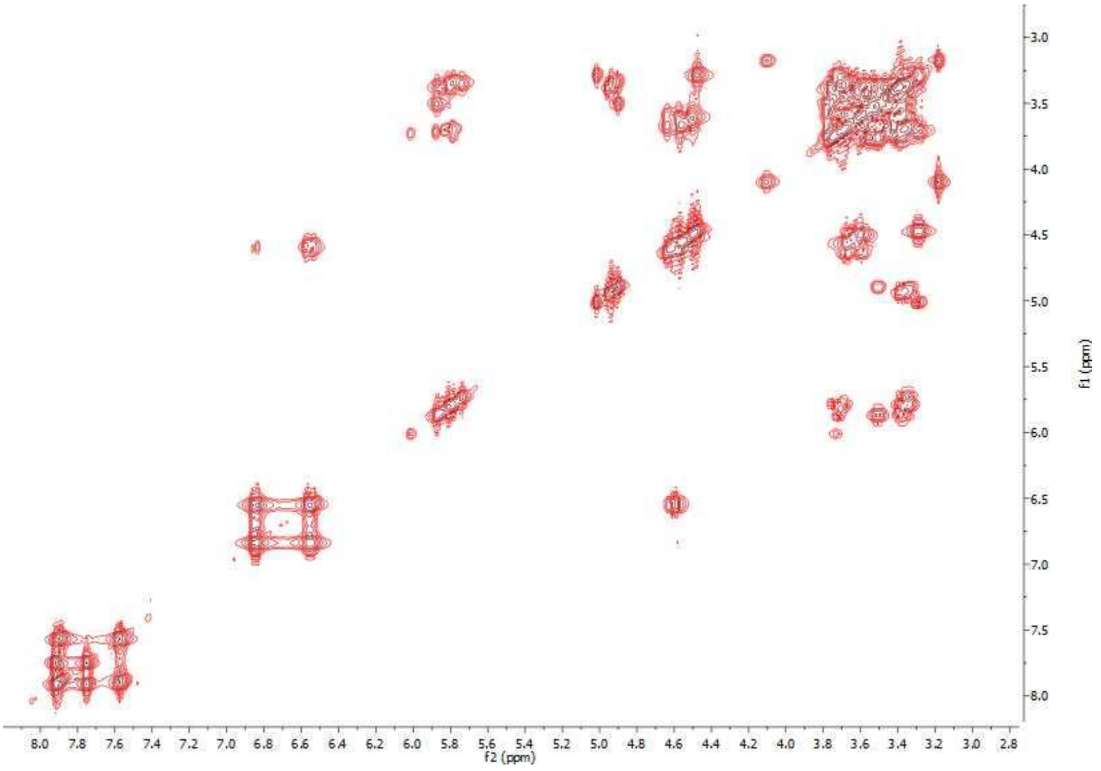

HSQC 2b

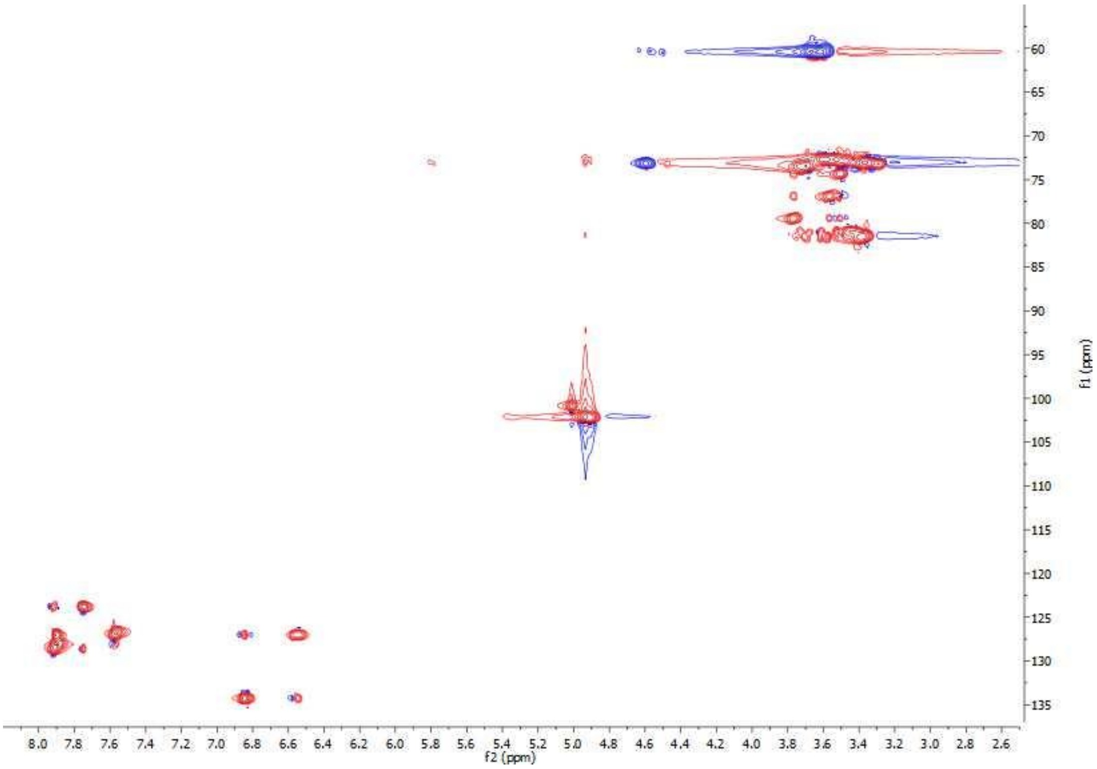

HMBC 2b

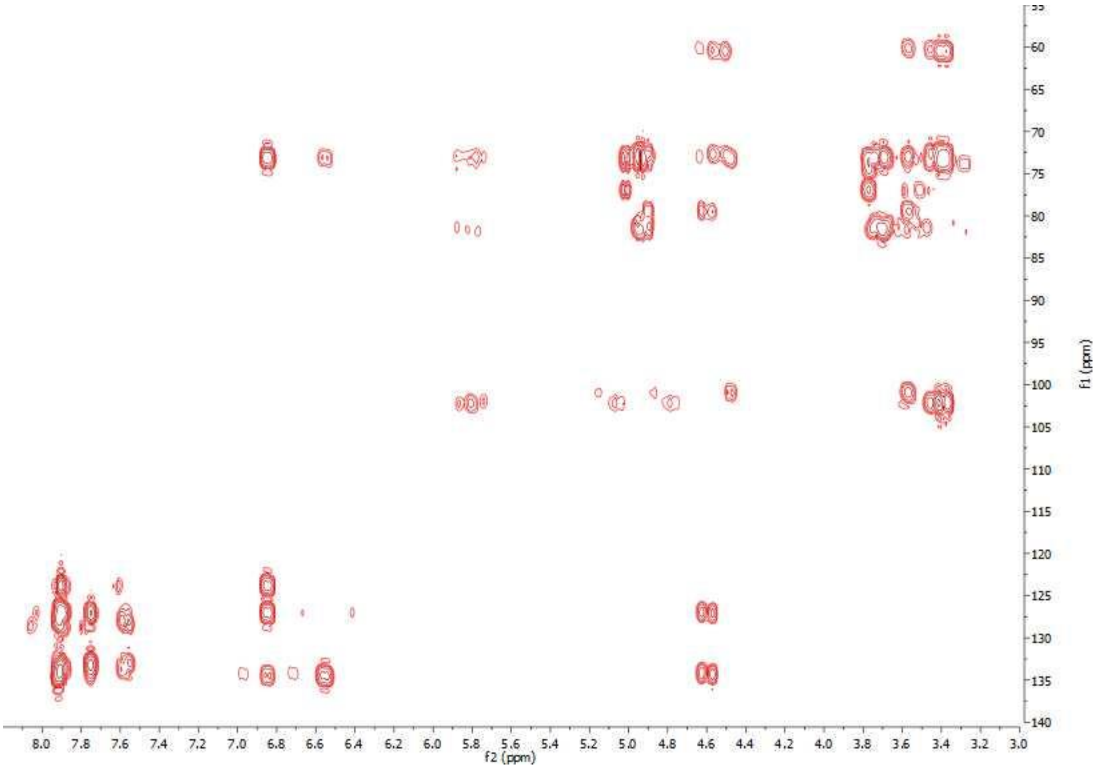

NMR spectra of 6^I-*O*-(3-(naphthalen-2-yl)prop-2-en-1-yl-γ-cyclodextrin (2c)
^{1}H 2c

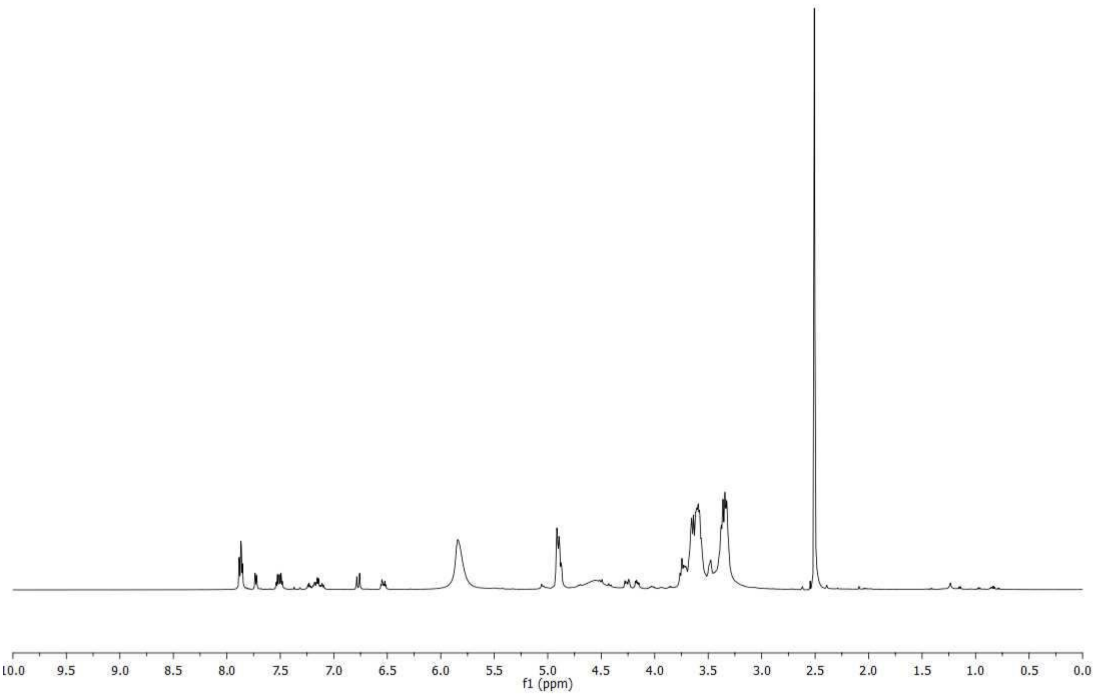

^{13}C 2c

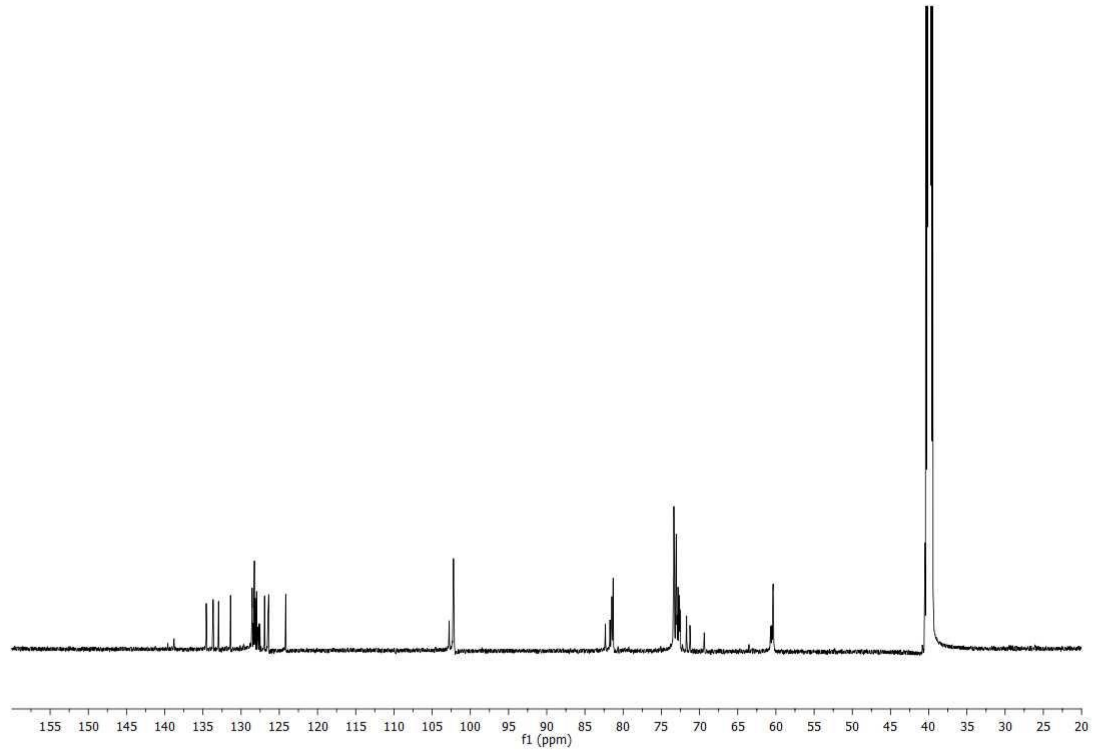

^{1}H-^{1}H – COSY 2c

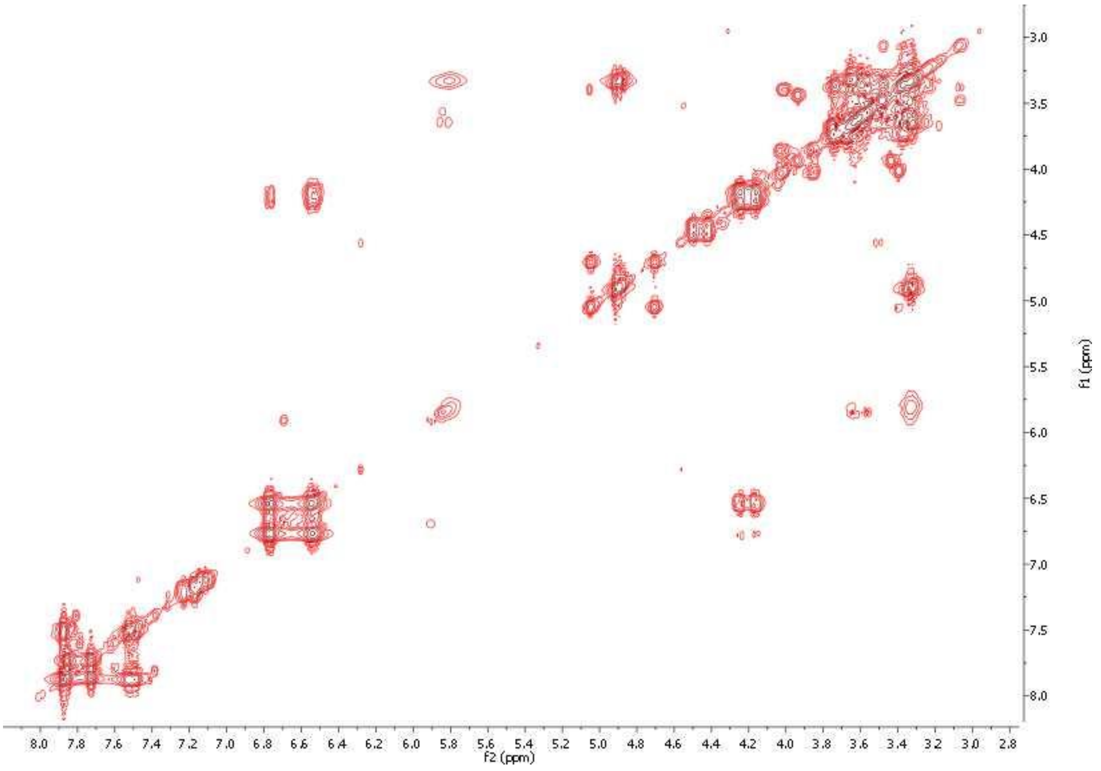

HSQC 2c

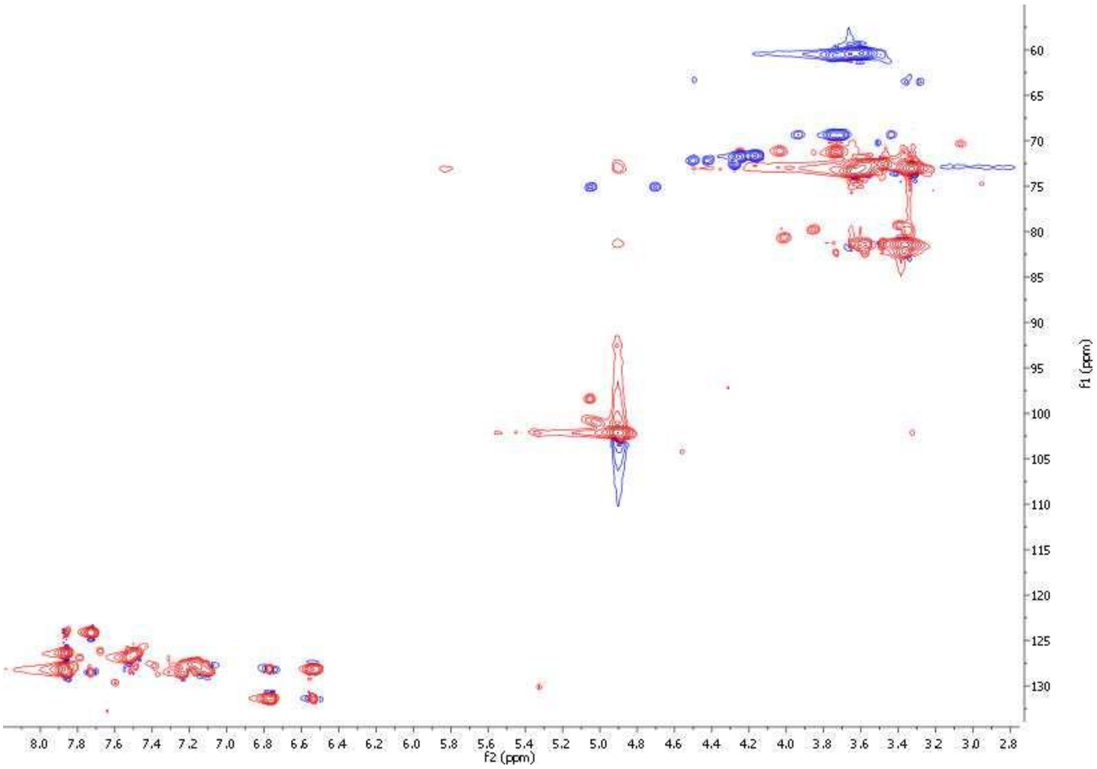

HMBC 2c

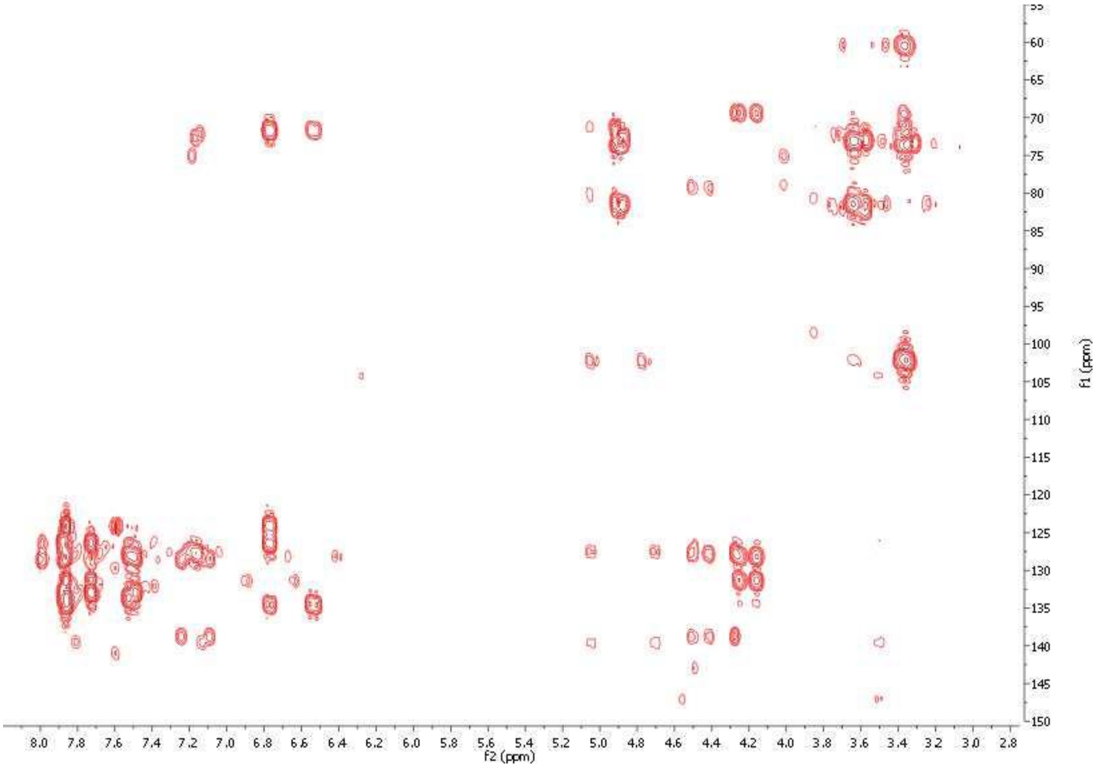

DLS

Correlation functions

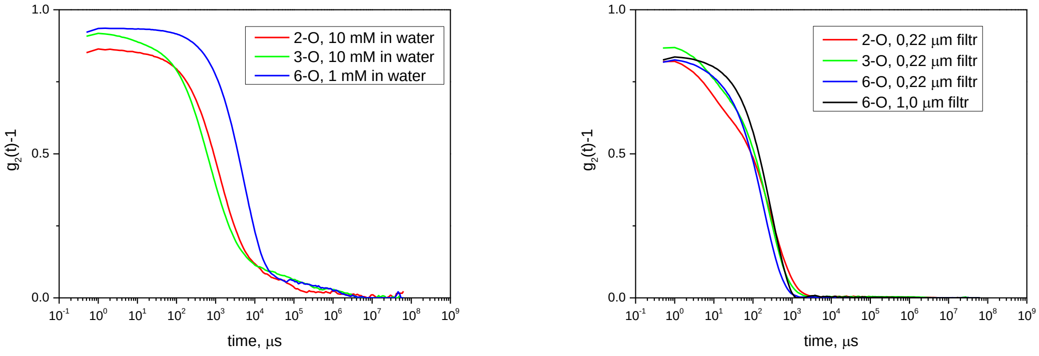

Figure S2: *Correlation functions for NA-γ-CD regioisomers water solutions before (left) and after (right) filtration*

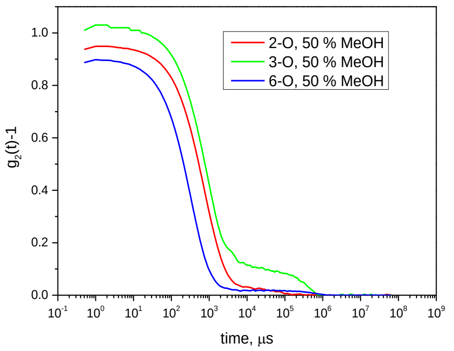

Figure S3: *Correlation functions for NA-γ-CD regioisomers in 50% MeOH (v/v)*

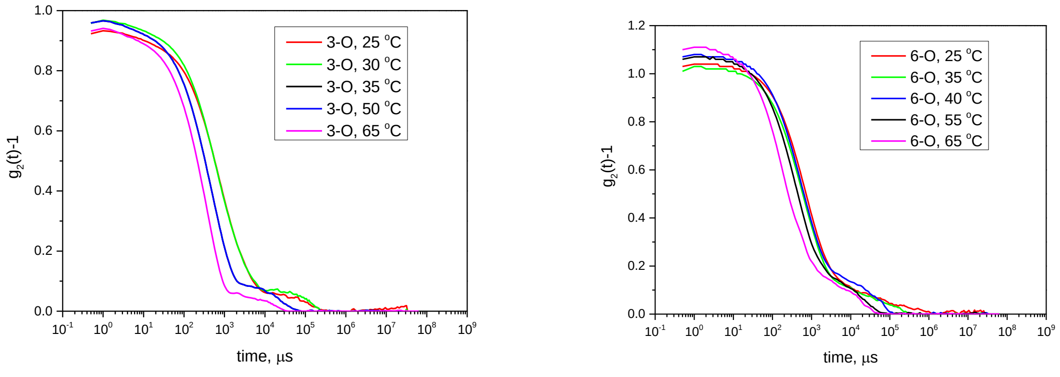

Figure S4: *Correlation function for temperature dependence of 3-O- and 6-O- regioisomers.*

Scattering intensity

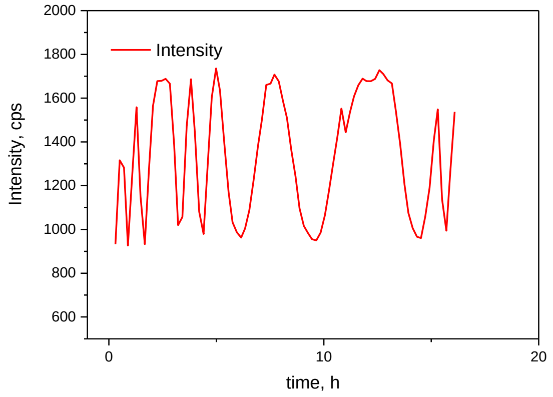

Figure S5: Time dependence of scattering intensity after filtration for the 3-O- isomer

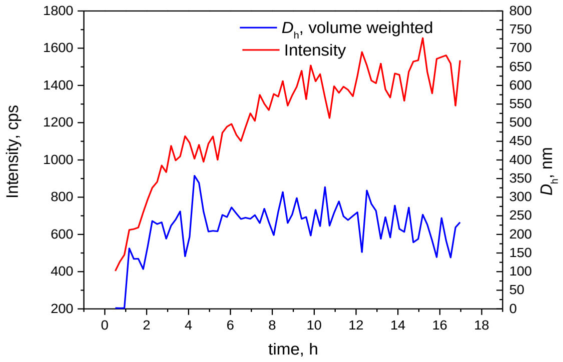

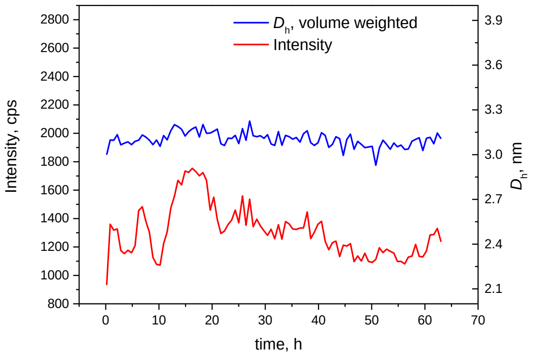

Figure S6: Time dependence of scattering intensity and volume-weighted Dh after filtration for the 6-O- (left) and 2-O- (right) isomer

γ-CD solution behavior

To obtain reference results, we also investigated the behavior of different concentration solutions of γ-CD in water (100, 50, 10, 1 mM). These samples prepared by serial dilution formed 1000 nm, 700 nm, 350 nm and 350 + 100 nm aggregates, respectively. Interestingly, the 10 mM sample of γ-CD prepared by direct dissolving of a solid compound contained just 1–2 nm particles according to its distribution function. The experiments were performed repeatedly, and the results are reproducible. Another series of γ-CD samples was prepared by

the dilution row again, but after that, the samples were stirred in a closed vial for 10 min at 120 °C, which led to formation of aggregates with sizes of 800 nm, 350 nm, 1.6, and 1.5 nm at 100, 50, 10, and 1 mM concentrations, respectively. Therefore, γ-CD aggregates survive in a large range of dilutions of the solution but are destroyed by boiling. However, Coleman [11] and Puskás [12] reported a different behavior of γ-CD solution. Puskás investigated aggregation properties of native CDs in water. While α-CD and β-CD showed similar results, forming primarily about 2 nm particles, γ-CD (1.0 wt% ~ 7.8 mM) formed aggregates with a broad size distribution from 50 to 600 nm. Coleman observed γ-CD (25 g/L ~19 mM) to form 200 nm aggregates. Occasionally, we also observed aggregates in 10 mM γ-CD prepared by the direct dissolving of a solid sample. However, in most cases, no aggregates of γ-CD were observed using this procedure. On the other hand, Messner et al. [13] summarized in their review that in ca. 10 mM aqueous solutions of native CDs the mass fraction of aggregated CD molecules is less than 1% (12 mM α-CD – 0.8% [14], 10 mM β-CD – 0.0011% [15-16], 12 mM γ-CD – 0.02% [17]). This means that the behavior of a solution of native γ-CD depends significantly on the method of its preparation. We can conclude that many factors are influencing the formation of aggregates (temperature, filtration, dilution, etc.) which limit the reproducibility of the measurements.

References

1. Bláhová, M.; Bednářová, E.; Řezanka, M.; Jindřich, J. *J. Org. Chem.* **2013**, *78*, 697–701.
2. Feng, Q.; Song, Q. *J. Org. Chem.* **2014**, *79*, 1867–1871.
3. Bouziane, A.; Hélou, M.; Carboni, B.; Carreaux, F.; Demerseman, B.; Bruneau, C.; Renaud, J.-L. *Chem. – Eur. J.* **2008**, *14*, 5630–5637.
4. Bulman Page, P. C.; Moore, J. P. G.; Mansfield, I.; McKenzie, M. J.; Bowler, W. B.; Gallagher, J. A. *Tetrahedron* **2001**, *57*, 1837–1847.
5. Zhang, K. *J. Struct. Biol.* **2016**, *193*, 1–12.
6. Schneider, C. A.; Rasband, W. S.; Eliceiri, K. W. *Nat. Methods* **2012**, *9*, 671–675.
7. McPhail, D.; Cooper, A. *J. Chem. Soc. Faraday Trans.* **1997**, *93*, 2283–2289.
8. Martinez, J. C.; Murciano-Calles, J.; S., E.; Iglesias-Bexiga, M.; Luque, I.; Ruiz-Sanz, J. In *Applications of Calorimetry in a Wide Context - Differential Scanning Calorimetry, Isothermal Titration Calorimetry and Microcalorimetry*; Elkordy, A. A., Ed.; InTech, 2013; pp. 74–104.
9. De Greef, T. F. A.; Smulders, M. M. J.; Wolffs, M.; Schenning, A. P. H. J.; Sijbesma, R. P.; Meijer, E. W. *Chem. Rev.* **2009**, *109*, 5687–5754.
10. Scherman, O. A.; Ligthart, G. B. W. L.; Sijbesma, R. P.; Meijer, E. W. *Angew. Chem. Int. Ed.* **2006**, *45*, 2072–2076.
11. Coleman, A. W.; Nicolis, I.; Keller, N.; Dalbiez, J. P. *J. Incl. Phenom. Mol. Recognit. Chem.* **1992**, *13*, 139–143.
12. Puskás, I.; Schrott, M.; Malanga, M.; Szente, L. *J. Incl. Phenom. Macrocycl. Chem.* **2012**, *75*, 269–276.
13. Messner, M.; Kurkov, S. V.; Jansook, P.; Loftsson, T. *Int. J. Pharm.* **2010**, *387*, 199–208.
14. González-Gaitano, G.; Rodríguez, P.; Isasi, J. R.; Fuentes, M.; Tardajos, G.; Sánchez, M. *J. Incl. Phenom. Macrocycl. Chem.* **2002**, *44*, 101–105.
15. Wu, A.; Shen, X.; He, Y. *J. Colloid Interface Sci.* **2006**, *302*, 87–94.
16. Wu, A.; Shen, X.; He, Y. *J. Colloid Interface Sci.* **2006**, *297*, 525–533.
17. Szente, L.; Szejtli, J.; Kis, G. L. *J. Pharm. Sci.* **1998**, *87*, 778–781.
